# MS-proteomics provides insight into the host responses towards alginate microspheres

**DOI:** 10.1016/j.mtbio.2022.100490

**Published:** 2022-11-11

**Authors:** Abba E. Coron, Davi M. Fonseca, Animesh Sharma, Geir Slupphaug, Berit L. Strand, Anne Mari A. Rokstad

**Affiliations:** a The Norwegian Biopolymer Laboratory (NOBIPOL), Department of Biotechnology and Food Science, Norwegian University of Science and Technology (NTNU), N-7491, Trondheim, Norway; b Department of Clinical and Molecular Medicine, NTNU, N-7491, Trondheim, Norway; c Proteomics and Modomics Experimental Core (PROMEC), NTNU and the Central Norway Regional Health Authority, N-7491, Trondheim, Norway; d Centre of Molecular Inflammation Research (CEMIR), Department of Clinical and Molecular Medicine, NTNU, N-7491, Trondheim, Norway; e Centre of Obesity, Clinic of Surgery, St. Olav's University Hospital, NO-7006, Trondheim, Norway

**Keywords:** Alginate hydrogel microspheres, Proteomics, Protein adsorption, Complement, Coagulation, Immune profiling

## Abstract

Protein adsorption to biomaterial surfaces is considered a determining factor for the host response. Here we detail the protein adsorption profiles of alginate hydrogel microspheres relevant for cell therapy using mass spectrometry (MS)-based proteomics. The investigated microspheres include sulfated alginate (SA), high G alginate (HiG), and poly-l-lysine coated alginate (AP), which previously have been shown to exhibit different inflammatory and fibrotic responses. The biological significance was assessed in lepirudin-anticoagulated human whole blood (hWB) by functional analysis of the acute-phase responses (complement and coagulation). Proteomic profiling revealed distinct signatures for the microspheres, wherein Ingenuity Pathway Analysis identified complement and coagulation as the top enriched canonical pathways. The levels of complement and coagulation activators and inhibitors were distinctly different, which was reflected in the functional hWB analyses: SA was highly enriched with inhibitory factors of complement and coagulation (e.g. C1 inhibitor, factor H, antithrombin-III, heparin cofactor 2), other heparin-binding proteins and factors promoting fibrinolysis (factor XII, plasma kallikrein), conforming to an anti-inflammatory and anti-fibrotic profile. HiG enriched moderate levels of complement inhibitors, conforming to a low-inflammatory and pro-fibrotic profile. AP showed the most prominent enrichment of complement activators (e.g. C3, properdin, C-reactive protein) and low levels of inhibitors, conforming to a pro-inflammatory and highly pro-fibrotic profile. In conclusion, the extensive enrichment of inhibitory acute-phase proteins on SA could be a determining factor for its reduced host response. The interactions between the plasma proteins and hydrogel surfaces shown herein point to proteomics as an important supplement to existing *in vitro* and *in vivo* methods for designing biocompatible alginate-based hydrogels.

## Highlights

• Mapping the protein adsorption profiles on alginate microspheres using mass spectrometry-based proteomics.
• Proteomic profiles conform to responses in human whole blood.
• Differential enrichment of acute-phase proteins.
• Sulfated alginate selectively binds inhibitors of complement and coagulation.
• Sulfated alginate selectively binds factors promoting fibrinolysis.

## Graphical abstract

**Figure:**
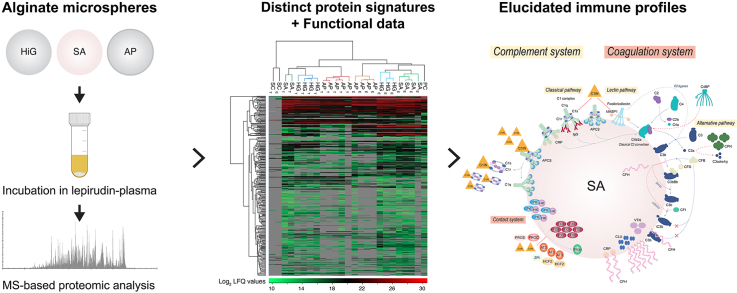

## 1 Introduction

The use of alginate hydrogel microspheres for immunoisolation in cell therapy holds great promise for treating various medical conditions, e.g. type 1 diabetes or acute liver failure, mitigating the need for systemic immunosuppressive treatment after graft transplantation [[1], [2], [3]]. However, implantation of biomaterials may result in acute and chronic inflammatory responses that lead to fibrotic tissue development, as part of the foreign body response [4]. For microspheres used in cell therapy, pericapsular fibrotic overgrowth (PFO) constitutes a major challenge, in which immune cells (neutrophils, macrophages) and fibroblasts hinder the diffusion of nutrients and oxygen, thus compromising the viability and function of the encapsulated cells [5,6]. Insight into the mechanistic cues associated with PFO will advance the development of high-performance, biocompatible materials for cell and tissue transplantation, with further impact on diagnostic sensors and implants. The initial protein adsorption to implanted biomaterials is considered a determining factor for the subsequent host responses [[4], [7], [8]]. Here, we investigate the protein adsorption profiles of three types of alginate microspheres that have previously been used or are promising candidates for clinical transplantation [1,9,10], which we recently assessed in terms of inflammatory and fibrotic potentials using *in vitro* and *in vivo* models [10]. Proteomics could serve as a complementary method to understand the initial protein adsorption important for the design and selection of alginate microspheres with reduced host responses.

Alginate hydrogels are ionically crosslinked networks (e.g. with Ca^2+^, Sr^2+^, Ba^2+^) of linear, anionic polysaccharides that consist of (1,4)-linked α-l-mannuronate (M) and β-d-guluronate (G) residues. The hydrogel is formed under physiological conditions, and the high water content (98–99%) allows for rapid diffusion of nutrients and oxygen to encapsulated cells, ensuring cell viability and function. Although generally regarded as biocompatible, the detailed design of alginate-based microspheres ultimately determines the onset of PFO and the long-term function of encapsulated cells *in vivo* [2,5]. Microspheres that are prone to PFO include the widely studied high G alginate microbeads [1,2,10,11], where the PFO response is exacerbated by coating with polyamines (e.g. poly-l-lysine; PLL) [2,12,13]. The prominent PFO response of PLL-coated microspheres [2,12] can be ascribed to surface-deposited complement C3, leading to leukocyte adhesion and subsequent induction of pro-inflammatory cytokines [[14], [15], [16]]. Several studies have demonstrated the involvement of complement activation/deposition (including complement C3) on biomaterial surfaces in promoting leukocyte activation/adhesion [[14], [15], [17], [18], [19], [20]], pro-inflammatory cytokine induction [[14], [15], [16], [17]] as well as inflammatory cell recruitment or fibrotic tissue formation after implantation [[17], [19]]. Mitigation of the PFO response has been achieved by modulating microsphere composition using intermediate G alginates [10,11,21] or chemically modified alginates [2,10,22,23]. Alginates modified by chemical sulfation have been shown to display anti-inflammatory properties [10,24,25] and bind growth factors [24,26] and anti-complement factor H [25]. In a recent study [10], we reported minimal PFO for microbeads containing a mixture of sulfated alginate and high G alginate (SA) using the immunocompetent C57BL/6JRj mouse model. Contrastingly, PFO was found on high G alginate microbeads (HiG) and to an even larger extent on PLL-coated high G alginate microbeads (AP). In detail, the anti-fibrotic SA as well as the fibrotic HiG induced overall low cytokine and complement responses *in vitro* and minimal C3 deposition *in vivo*. The highly fibrotic AP induced higher pro-inflammatory cytokine responses and significant complement activation *in vitro* and displayed marked C3 deposition *in vivo*. Fibrin(ogen) deposition on the microspheres was found to coincide with PFO, further pointing to distinct protein deposition as a potential contributing factor to PFO. In the current proteomic study, we further analyse these microspheres with respect to protein adsorption in human plasma and link the proteomic findings to the inflammatory and fibrotic potentials of the different materials.

Mass spectrometry (MS)-based proteomic analysis represents an efficient and highly sensitive approach to identify protein adsorption profiles on biomaterial surfaces [7], and thus provides an additional means to predict biological performance and potentially reduce *in vivo* experimentation. To date, there is a limited number of proteomic studies that have addressed the adsorption of plasma or serum proteins to hydrogels [20,27,28], and neither of these encompassed alginate-based hydrogels or microspheres. Previous studies using MS-based proteomics have linked protein adsorption to the immune reactivity [20] or fibrotic tissue development [27] of poly(ethylene glycol) (PEG) hydrogels. The latter revealed an initial adsorption of acute-phase proteins for highly fibrotic PEG hydrogels explanted from mice [27]. More generally, there are few studies that directly correlate biomaterial protein adsorption using proteomics with *in vivo* performance [7,29]. In a collection of studies (e.g. Refs. [30,31]), Romero-Gavilán and co-workers characterise distinct protein layers on titanium-based implants using human serum. The authors correlate the differentially adsorbed proteins (mainly complement related) to the level of fibrotic tissue development and osseointegration in rabbits. Using the same animal model and calcium-doped materials, they also found distinct enrichment of proteins associated with coagulation, inflammation, and osteogenic functions, which were linked to the materials’ regenerative potential *in vivo* [32]. Buck et al. showed that surface functionalisation of poly(ether ether ketone) (PEEK) implants significantly altered the adsorption of serum proteins (predominantly acute-phase proteins and apolipoproteins), where implantation into rats revealed similar levels of osseointegration despite different macrophage responses *in vitro* [33]. The protein adsorption to nanoparticles and its effect on biological performance *in vivo* has also been described [[34], [35], [36]].

Protein adsorption is a complex and dynamic process in which proteins attach and detach depending on the properties of the surface, proteins, and surrounding solution [37,38]. Protein adsorption has been linked to the biomaterials' chemical properties [39], surface charge [8] and topography [40]. In addition to non-covalent surface interactions (e.g. electrostatic, hydrophobic) that can result in conformational changes potentially affecting the bioactivity of adsorbing proteins, the proteins may also *react* with the biomaterial surface as well as surface-associated proteins. These features are well-recognised for the complement and coagulation proteins, which are critical components of the acute-phase responses in host defence [41]. Adsorbed complement activators and inhibitors have been suggested to be strong indicators for the biocompatibility of biomaterial surfaces [8,42]. The current study was designed to preserve the proteolytic cascades of complement and coagulation by using the anticoagulant lepirudin. This anticoagulant does not interfere with the complement proteins or coagulation proteins upstream of thrombin, in contrast to heparin which interferes with both systems [43,44]. In addition, the anticoagulants citrate and EDTA, commonly used in studies assessing the binding of plasma proteins to biomaterials, also interfere with the reactivity of the complement and coagulation systems by chelating calcium. Lepirudin has been used as an anticoagulant in several studies on inflammatory responses in whole blood, including studies on dialysis membranes [45], glucose sensors [46], polyvinyl chloride surfaces [47], and alginate microspheres [[14], [15], [16],^48^].

Here, we present the first study on LC-MS/MS-based quantitative profiling of plasma proteins adsorbed to alginate hydrogel microspheres using a physiologically relevant human plasma model. Three types of microspheres were selected based on their different PFO responses and inflammatory potentials known from previous work [10]: HiG (low-inflammatory and fibrotic), SA (low-inflammatory and anti-fibrotic) and AP (pro-inflammatory and highly fibrotic). Unique protein signatures were identified for the different alginate microspheres. Immune profiles of the microspheres were detailed by combining proteomics and functional studies on initial inflammatory responses, with a particular focus on the acute-phase proteins of the complement and coagulation systems. This study represents a novel approach for elucidating proteomic profiles of alginate-based hydrogels that gives insight into the host-material interactions at the protein level, with potential relevance to PFO.

## 2 Materials and methods

### 2.1 Materials

Ultra-pure (UP) sodium alginates (endotoxin ≤43 EU/g) were from Novamatrix (Sandvika, Norway). UP-low-viscosity high G (LVG) alginate (68% guluronate [G], duplet fraction [F_GG_] ​= ​0.57, triplet fraction [F_GGG_] ​= ​0.53, average G-block length [N_G ​> ​1_] ​= ​16, weight average molecular weight [Mw] ​= ​237 ​kDa) was used as gelling alginate. UP-medium-viscosity high G (MVG) alginate (66% G, F_GG_ ​= ​0.55, F_GGG_ ​= ​0.50, N_G ​> ​1_ ​= ​13, weight average Mw ​= ​235 ​kDa) was used to produce sulfated alginate. Determination of the alginate chemical composition by H^1^-NMR [49,50] and molecular weight by SEC-MALLS [51] has previously been described. Preparation of alginates and alginate microspheres included analytical grade CaCl_2_, BaCl_2_, NaCl, and formamide from Merck (Darmstadt, Germany). Poly-l-lysine (PLL) hydrochloride (Mw ​= ​15–30 ​kDa) and chlorosulfonic acid (99%) were from Sigma-Aldrich (St. Louis, MO, USA). D(-)-Mannitol was from VWR International BVBA (Leuven, Belgium) and non-pyrogenic sterile saline (0.9% NaCl) from B. Braun (Melsungen, Germany). Sulfated UP-MVG alginate was purified using Millistak+® CR40 activated carbon filter from Millipore (Billerica, MA, USA) and endotoxin-tested by QCL-1000™ Endpoint Chromogenic LAL Assay from Lonza (Walkersville, MD, USA). Human plasma and whole blood were anticoagulated with lepirudin (Refludan) from Celgene Europe (Boudry, Switzerland). Proteomic sample preparations employed the following (chemicals were at least pro analysis): urea, CHAPS hydrate, thiourea, DTT, ammonium bicarbonate, iodoacetamide, and solid-phase extraction disks (Empore C18, 47 ​mm) from Sigma-Aldrich; LC/MS grade: methanol, formic acid, acetonitrile, water, and Pierce trypsin from Thermo Fisher Scientific, USA; chloroform from VWR International S.A.S., France. For protein deposition studies by CLSM, FITC-conjugated polyclonal rabbit anti-human C3c (F0201) and C1q (F0254), including control antibody polyclonal rabbit-anti-mouse immunoglobulins (F0232), were from Dako (Glostrup, Denmark). Unconjugated polyclonal sheep anti-human FXII was from Nordic Diagnostica Service AB (HTI, Kungsbacka, Sweden), and secondary CF633-conjugated polyclonal donkey anti-sheep IgG was from Sigma-Aldrich. In the human whole blood experiment, assays used were enzyme-linked immunosorbent assay (ELISA) kit for Human Terminal Complement Complex (TCC) from Hycult Biotech (Uden, Netherlands), and ELISA kit Enzygnost® F1+2 monoclonal from Siemens Healthcare Diagnostics (Marburg, Germany). Low-activating polypropylene vials were from NUNC (Roskilde, Denmark). Glass control (BD vacutainer glass) was from Belliver Industrial Estate (Plymouth, UK), and PBS with CaCl_2_ and MgCl_2_ was purchased from Sigma-Aldrich.

### 2.2 Sulfation of alginate

Alginate was sulfated as previously described [24]. Briefly, chlorosulfonic acid was carefully added to a suspension of alginate (3.0 ​g) and formamide (120 ​mL) to a final concentration of 2.91% v/v. The mixture was incubated at 60 ​°C under continuous agitation for 2.5 ​h. Alginate was precipitated using cold acetone, centrifuged (10 ​°C, 3600×*g*, 7 ​min), redissolved in Milli-Q water and pH-neutralised. The alginate solution was dialysed against 100 ​mM NaCl, four times against Milli-Q water, and freeze-dried. The sulfur content of the alginate was determined to be 8.5% by high-resolution inductively coupled plasma mass spectrometry (HR-ICP-MS) at SINTEF, Trondheim, Norway. The degree of sulfation was estimated to be 0.83, as previously described [24]. Sulfated alginate was purified using an active carbon filter, and the level of endotoxins (LAL assay) was measured to 3.126 EU/mL.

### 2.3 Preparation of alginate microspheres

Three different microspheres were prepared using the same alginate formulations as in our recent study on PFO [10], comprising unmodified alginate (HiG), a mixture of 20% sulfated alginate and 80% unmodified alginate (SA), and unmodified alginate with PLL-coating (AP). Alginate solutions (5 ​mL) of 1.8% (w/v) were dripped into a gelling bath containing either 50 ​mM CaCl_2_ with 1 ​mM BaCl_2_ (HiG and SA) or 50 ​mM CaCl_2_ (AP). All formulations were made using an electrostatic droplet generator operated at 7 ​kV, with a flow rate of 10 ​mL/h and needle size of 0.4 ​mm [52]. All solutions were sterile filtered, and the microspheres were prepared under sterile conditions. Alginate and gelling solutions were dissolved in 0.3 ​M and 0.15 ​M mannitol, respectively, and pH-adjusted to 7.2–7.3. Alginate microbeads were left for 10 ​min after the last formed droplet and washed in 0.9% NaCl (30 ​mL). AP-microspheres were subsequently incubated in 0.1% PLL dissolved in 0.9% NaCl (25 ​mL, pH ​= ​7.35) for 10 ​min, and washed in 0.9% NaCl (30 ​mL). Each batch of microspheres was added 0.9% NaCl (50 ​mL) and aliquoted into samples containing 0.5 ​mL microspheres. Lastly, each aliquot was washed with 0.9% NaCl (2 ​× ​1 ​mL) and further aliquoted into samples containing 50 ​μL microspheres. Microsphere diameters (mean ​± ​SD of *n* ​= ​30) were measured to 579 ​± ​17 ​μm (HiG), 565 ​± ​35 ​μm (SA) and 538 ​± ​34 ​μm (AP).

### 2.4 Proteomic sample preparation

#### 2.4.1 Incubation of microspheres in human lepirudin-plasma

Alginate microspheres (50 ​μL) were incubated in pooled (*N* ​= ​7) lepirudin-plasma (300 ​μL) for 24 ​h at 37 ​°C under rotation, with five replicates for each type of microsphere. Human blood was anticoagulated by adding lepirudin (50 ​μg/mL), centrifuged (1880×*g*, 15 ​min), and harvested plasma stored at -80 ​°C. Control samples comprised microspheres (50 ​μL) incubated in 300 ​μL 0.9% NaCl (saline control) and a pooled plasma control sample (10 ​μL). Low-activating polypropylene vials were used for all samples. Microspheres were washed in 0.9% NaCl (2 ​× ​500 ​μL) to remove non-adsorbing proteins. Samples were stored in 0.9% NaCl (100 ​μL) at 4 ​°C. The storage solution was removed before analysis.

#### 2.4.2 Primary elution of adsorbed plasma proteins (E-fraction)

Microspheres and control samples were incubated in 2D-PAGE buffer (100 ​μL, 7.0 ​M Urea, 2.0 ​M thiourea, 2.5% CHAPS, 25 ​mM DTT) for 2 ​h on a rotary shaker (37 ​°C, 400 ​rpm), and eluates were transferred to new tubes. 2D-buffer incubation was repeated (5 ​min), and the respective eluates were pooled. Eluates were stepwise added methanol (800 ​μL), chloroform (200 ​μL) and water (600 ​μL) with intermittent vortexing, and then centrifuged (16 ​000×*g*, 15 ​min). The top aqueous layer was removed, and methanol (800 ​μL) was added, followed by vortexing and centrifugation (16 ​000×*g*, 60 ​min). The supernatant was removed, and the pellet with remaining solution was evaporated to dryness. 50 ​mM ammonium bicarbonate (Ambic; 100 ​μL) was added to the pellet, and samples were vortexed. Supernatants were stepwise added 0.5 ​M DTT (4 ​μL, 30 ​min), 0.2 ​M iodoacetamide (30 ​μL, 30 ​min in the dark), 0.5 ​M DTT (8 ​μL, 20 ​min), incubated overnight on a rotary shaker (37 ​°C, 400 ​rpm) in 12.5 ​ng/μL trypsin in 44 ​mM Ambic (100 ​μL), and evaporated to dryness.

#### 2.4.3 On-microsphere trypsination of residual plasma proteins (T-fraction)

Following the primary elution, microspheres were washed in 50 ​mM Ambic (200 ​μL) for 1 ​h on a rotary shaker (24 ​°C, 400 ​rpm) and further washed in 50 ​mM Ambic (2 ​× ​200 ​μL). Microspheres were added 50 ​mM Ambic (100 ​μL) and stepwise treated with DTT/iodoacetamide/DTT as described above. Samples were washed in 50 ​mM Ambic (200 and 100 ​μL, respectively), resuspended in 50 ​mM Ambic (100 ​μL), and incubated overnight on a rotary shaker (37 ​°C, 400 ​rpm) in 12.5 ​ng/μL trypsin in 44 ​mM Ambic (100 ​μL). Released tryptic peptides were transferred to new tubes. Microspheres were washed in 50 ​mM Ambic (50 ​μL), and residual released peptides were pooled with respective samples. Tryptic eluates were evaporated to dryness.

#### 2.4.4 Preparation of peptides in E- and T-fractions

Dried peptides were reconstituted in 0.1% formic acid in water (60 ​μL). Stage tip columns consisting of three C-18-filters were prewashed with methanol (3 ​× ​50 ​μL), centrifuged (1500×*g*, 3 ​min) for each wash, equilibrated with 0.1% formic acid in water (3 ​× ​100 ​μL), and centrifuged (1500×*g*, 2 ​min) for each equilibration step. Peptide samples were centrifuged (16 ​000×*g*, 25 ​min), supernatants loaded onto separate stage tip columns and centrifuged (1500×*g*, 4 ​min). Flow-through solutions were reloaded to stage tip columns and centrifuged (1500×*g*, 3 ​min). Stage tip columns were washed with 0.1% formic acid (2 ​× ​60 ​μL), centrifuged (1500×*g*, 3 ​min) for each wash, and flow-throughs were discarded. Peptides were eluted from the stage tip column using 0.1% formic acid in 70% acetonitrile (2 ​× ​40 ​μL), centrifuged (1500×*g,* 1 ​min) for each elution, and evaporated to dryness. Dried peptides were reconstituted in 0.1% formic acid in water (60 ​μL), vortexed, and agitated for 1–3 ​h (4 ​°C, 900 ​rpm). Samples were centrifuged (16 ​000×*g*, 15 ​min), and supernatants (30 ​μL) were transferred to MS-vials for LC-MS/MS analysis.

### 2.5 Liquid chromatography-tandem mass spectrometry (LC-MS/MS)

LC-MS/MS was performed on an EASY-nLC 1000 UPLC system (Thermo Scientific) interfaced with an Orbitrap Elite mass spectrometer (Thermo Scientific) via a Nanospray Flex ion source (Thermo Scientific). Peptides were injected onto an Acclaim PepMap100C18 trap column (75 ​μm i.d., 2 ​cm long, 3 ​μm, 100 ​Å, Thermo Scientific) and further separated on an Acclaim PepMap100C18 analytical column (75 ​μm i.d., 50 ​cm long, 2 ​μm, 100 ​Å, Thermo Scientific) using a 120-min multi-step gradient (3 ​min 2%–6% B, 92 ​min 6%–30% B, 5 ​min 30%–40% B, 5 ​min 40%–100% B and 15 ​min at 100% B; where B is 0.1% formic acid in acetonitrile and A is 0.1% formic acid in water) at 250 ​nL/min. Peptides were analysed in positive ion mode under data-dependent acquisition using the following parameters: Electrospray voltage 2.5 ​kV, CID fragmentation with normalised collision energy 35 and 10 ​ms activation time. Each MS scan (400–1600 ​*m*/*z*, 1 ​*m*/*z* isolation width, profile) was acquired at a resolution of 120 ​000 FWHM in the Orbitrap analyser, followed by rapid MS/MS scans (2 ​*m*/*z* isolation width, centroid) triggered for the 15 most intense ions, with a 40 ​s dynamic exclusion and analysed in the linear ion trap. Charge exclusion was set to unassigned, and 1.

### 2.6 Proteomic data analysis

**Fig. 1:**
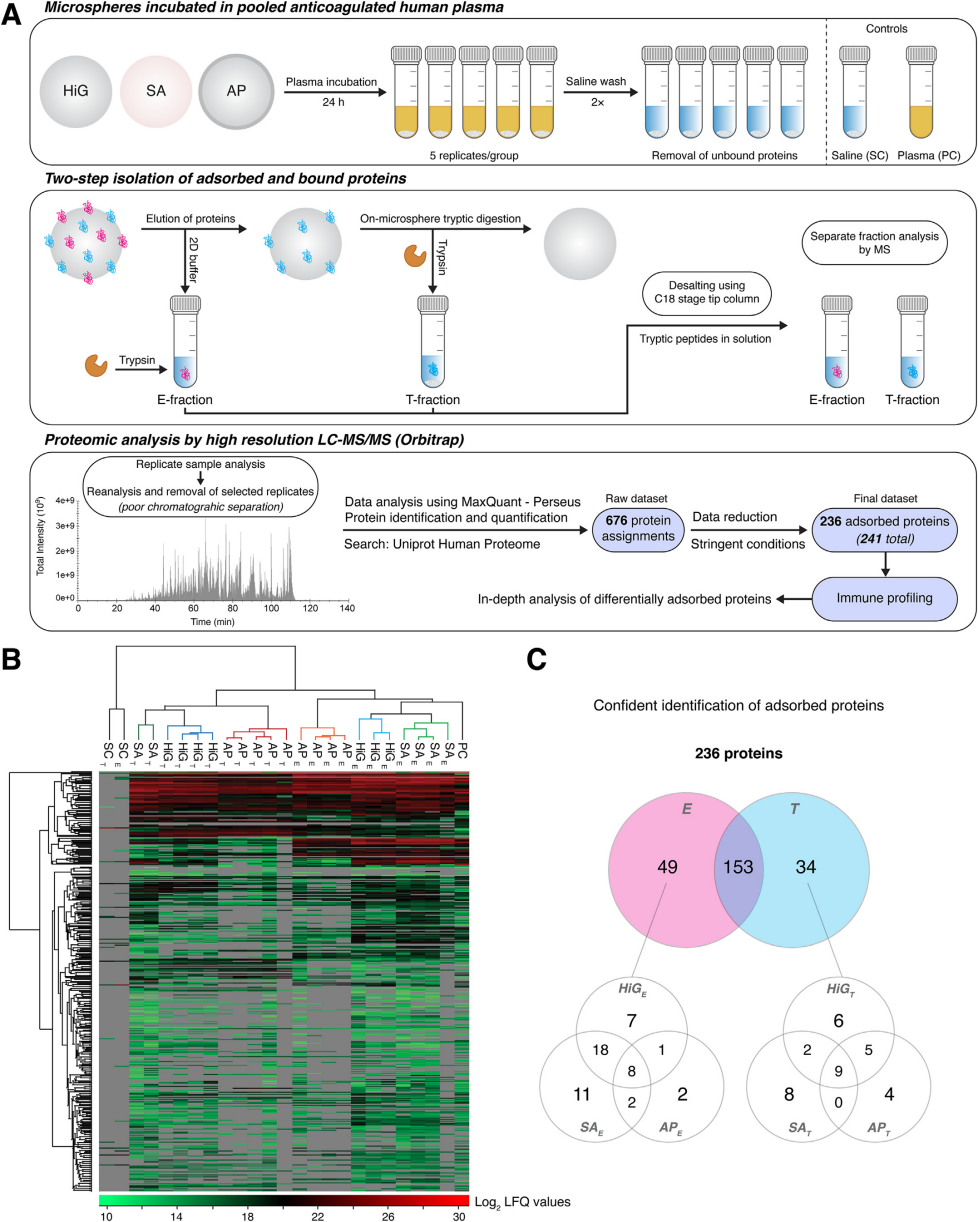
Proteomic analysis of human plasma proteins adsorbed to alginate microspheres after incubation in lepirudin-plasma (24 ​h). (**A**) Workflow of the proteomic study using liquid chromatography-tandem mass spectrometry (LC-MS/MS). Identification and quantification of plasma proteins adsorbed to alginate microspheres HiG, SA, and AP, separated into eluted (E)- and on-microsphere trypsinated (T)-fractions. Control samples included saline (SC; microspheres in saline) and plasma (PC; unfractionated lepirudin-plasma). (**B**) Heatmap showing identified protein adsorption profiles for the alginate microspheres. Pairwise Pearson's correlation coefficient was used on the raw dataset to calculate the Euclidean distance for hierarchical clustering of proteins (*rows*) and samples (*columns*). Protein abundances are coloured based on log_2_ label-free quantification (LFQ) intensity values, indicated by a colour scale bar (*bottom*). Not detected proteins are indicated in grey. (**C**) Venn diagrams show 236 differentially adsorbed proteins between microsphere fractions (E vs T) and between microsphere types. (For interpretation of the references to colour in this figure legend, the reader is referred to the Web version of this article.)

LC-MS/MS data were initially collected from 5 replicates for each type of alginate microsphere, one saline control and one plasma control. A selection of samples was re-injected into the LC/MS due to some replicates displaying unsatisfactory chromatographic separation (namely, few and broad peaks). Initial data analysis (similar to the one described below) was performed for all collected data, i.e. initial injections and re-injections. This initial analysis culminated in the heatmap of z-scaled median transformed label-free quantification (LFQ) values with base 2 of the sample population, Supplementary Fig. S1. Considering the chromatographic quality and grouping of samples, we removed outliers resulting in the following number of replicates for each microsphere fraction: 3 (HiG_E_), 4 (HiG_T_), 4 (SA_E_), 2 (SA_T_), 4 (AP_E_) and 5 (AP_T_); our analyses and findings are based on these. Preview 2.3.5 (Protein Metrics Inc. https://www.proteinmetrics.com) was used to determine optimal search criteria for the raw files. These parameters were plugged into MaxQuant [53] v 1.5.8.3, which utilises the MaxLFQ algorithm [54] mapping the spectra over Human canonical proteome including isoforms (downloaded in March 2017 [55]). The following search parameters were used: enzyme specified as trypsin with a maximum of 2 missed cleavages allowed, deamidation of asparagine/glutamine, oxidation of methionine, N-terminal acetylation as variable- and carbamidomethylation of cysteine as fixed modification. Mass tolerance for FTMS-MS and ITMS-MS/MS was set to 20 ​ppm and 0.5 ​Da, respectively, with false discovery rate (FDR) ​< ​0.01 (high confidence) for peptide spectra matches (PSM), peptide as well as protein group identification. LFQ values for identified protein groups were log-transformed with base 2. Filtered technical replicates were collapsed to their median value, each representing a biological replicate for a set condition. Proteins included in the final dataset were identified by two or more peptides at a fixed FDR protein level of 1.0%. The cut-off for log_2_-transformed LFQ values was set to 16.0 as per the MS-equipment discovered noise-sensitivity level. Proteins assigned with negative PSM scores, identified only by a site, as well as duplicates and known contaminants such as keratins, were removed from the dataset. Protein groups identified in >70% of replicates of at least one group were retained. For the cluster analysis, log_2_ LFQ values were z-scaled (except Fig. 1B). The obtained values are relative to the median of the whole expression profile and scaled to variation reflecting relative over- and under-expression. Raw file clustering was based on column-wise Z-scaling. The Euclidean distance between the expression vector was used for the hierarchical clustering (http://coxdocs.org/doku.php?id=perseus:user:activities:MatrixAnalysis:ClusteringPCA:HierarchicalCluster) using a k-means algorithm for pre-processing and average linkage for grouping. Median log_2_ LFQ intensities representing sample types were presented to Ingenuity® Pathway Analysis (IPA; QIAGEN Inc., https://www.qiagenbioinformatics.com/products/ingenuity-pathway-analysis [56]) to identify top canonical pathways, using an intensity threshold of 16.0. Raw files have been deposited to the ProteomeXchange Consortium [57] via the PRIDE partner repository with the dataset identifier PXD009135 (https://www.ebi.ac.uk/pride/archive/projects/PXD009135) and Notur/NorStore Project NN9036K/NS9036K, respectively.

### 2.7 Adsorption of plasma proteins to alginate microspheres evaluated by confocal laser scanning microscopy (CLSM)

Alginate microspheres (50 ​μL) were incubated in pooled (*N* ​= ​4) lepirudin-plasma (300 ​μL) for 24 ​h at 37 ​°C under rotation and subsequently washed in 0.9% NaCl (2 ​× ​500 ​μL). Microspheres were stained for complement factors C1q (polyclonal rabbit anti-human C1q/FITC, 50 ​μg/mL), C3c (polyclonal rabbit anti-human C3c/FITC, 50 ​μg/mL), or coagulation FXII by unconjugated polyclonal sheep anti-human FXII (50 ​μg/mL) and secondary staining with polyclonal donkey anti-sheep IgG/CF633 (20 ​μg/mL). Controls for non-specific antibody binding were polyclonal rabbit-anti-mouse immunoglobulins/FITC (50 ​μg/mL) and polyclonal donkey anti-sheep IgG/CF633 (20 ​μg/mL). All incubations were performed for 30 ​min (37 ​°C, under rotation) with subsequent washing in 0.9% NaCl (2 ​× ​500 ​μL). Samples were stored in 0.9% NaCl (200 ​μL) before analysis and protected from light. Protein deposition was assessed by CLSM (Zeiss LSM 510 Meta, Carl Zeiss MicroImaging GmbH, Göttingen, Germany) using a C-Apochromat 10 ​× ​/0.45w objective and pinhole of 30.1 ​μm. 2D images were captured by optical cross-sections through the microsphere equator. 3D images were constructed from sections through the entire microsphere using z-stacks and ImageJ software (National Institutes of Health, New York, USA). An argon laser with an excitation wavelength of 488 ​nm at 20% laser power, emission at 500–550 ​nm, and gain 515, were used for FITC-conjugated antibodies. A helium-neon laser with an excitation wavelength of 633 ​nm at 50% laser power, emission over 650 ​nm, and gain 530, were used for the CF633-conjugated antibody.

### 2.8 Complement and coagulation reactivity of microspheres in human whole blood

Initial complement and coagulation responses to microspheres upon blood contact were evaluated using lepirudin-anticoagulated human whole blood, as previously described [44] and with modifications for microspheres [14]. In brief, aliquoted samples of either microspheres (50 ​μL) in 0.9% NaCl (50 ​μL) or controls (baseline, PBS [background] and glass) were added 0.9% NaCl (100 ​μL). All samples were further added PBS with CaCl_2_ and MgCl_2_ (100 ​μL). Blood was collected from healthy donors (*N* ​= ​5) in low-activating polypropylene vials containing the anticoagulant lepirudin (50 ​μg/mL). Samples were incubated in blood (500 ​μL) for 4 ​h (37 ​°C, continuous rotation), and EDTA was added to a final concentration of 10 ​mM to inactivate the complement and coagulation responses. For the baseline sample, EDTA-inactivation was performed prior to blood incubation. Samples were centrifuged (4 ​°C, 1880×*g*, 15 ​min), plasma was harvested and stored at -20 ​°C before analysis. Complement activation was measured by the enzyme-linked immunosorbent assay (ELISA) kit for detecting soluble TCC. The assay was performed in accordance with the provided protocol. Coagulation activation was assessed by the level of prothrombin cleavage fragment F1+2 (PTF1.2) by ELISA kit Enzygnost® F1+2, monoclonal. Analysis was performed following the producer's protocol but included modifications to the plasma dilutions (1:10–1:1000).

### 2.9 Statistical analyses

In the proteomic analysis, plasma proteins on microspheres were statistically analysed with R [58], using log_2_-transformed LFQ values for group comparisons. For enrichment against the plasma control, a one-sample Student's T-test [59] was employed with the assumption that the null values were 0. Protein groups missing from all microspheres and identified in the plasma control were categorised as *repelled* by microspheres with values 0. Values only represented in one group were also set as 0 in subsequent Benjamini-Hochberg corrections [60]. Only protein groups with FDR <0.1 were considered for further analysis. Protein hydrophobicity and isoelectric points (pI) were calculated using the Peptides software package [61] with the Kyte-Doolittle scale for hydrophobicity and Bjellqvist scale for pI.

For the human whole blood experiment, repeated measures one-way ANOVA with Geisser-Greenhouse correction and Tukey's multiple comparison test was used to define statistical differences between selected sample groups. Data were log-transformed before analysis due to low sample numbers (*N* ​= ​5). Differences between sample groups were considered significant at p ​< ​0.05.

### 2.10 Ethics

The use of blood and plasma from volunteers has been granted by the Regional Ethics Committee of Mid-Norway under REC Central (REK2009/2245), following their recommended guidelines.

## 3 Results

### 3.1 Proteomic analysis of human plasma proteins adsorbed to alginate microspheres

Adsorbed plasma proteins were identified by high-resolution LC-MS/MS as outlined in Fig. 1A. Label-free quantification of peptides (E-/T-fractions and controls) identified a total of 676 protein groups with relative protein abundances given as log_2_ LFQ intensity values. Hierarchical clustering of quantified proteins revealed distinct protein adsorption profiles for alginate microspheres HiG, SA, and AP, and the E- and T-fractions thereof (Fig. 1B). Proteins quantified by replicate analysis of each microsphere type invariantly clustered together, indicating high reproducibility of our method. For confident identification of proteins, the initial dataset was further processed using strict conditions: log_2_ LFQ value cut-off at 16.0, peptide count of at least 2 for identified proteins and the removal of peptides assigned with negative PSM-scores, duplicates and known contaminants. This resulted in a final dataset of 241 confidently identified protein groups (Supplementary Table S1), wherein 236 proteins were identified on the alginate microspheres. In the unfractionated plasma control, 128 proteins were identified and 5 of these proteins were not found on the microspheres. Hence, a total of 113 proteins were exclusively identified on the microspheres and thus enriched above their detection level in unfractionated plasma in our LC-MS/MS setup. A higher number of proteins were identified on microspheres HiG and SA compared to AP: HiG (167 ​E-/149 ​T-proteins), SA (182 ​E-/139 ​T-proteins) and AP (120 ​E-/114 ​T-proteins). As shown in Fig. 1C, several adsorbed plasma proteins were uniquely distributed between the E- and T-fractions. Of the 236 adsorbed proteins, 49 proteins were only identified in the E-fractions and 34 proteins were only identified in the T-fractions. It is reasonable to anticipate that the proteins uniquely identified in the T-fractions represent the proteins that bind most strongly to the microspheres.

### 3.2 Enrichment of plasma proteins on alginate microspheres

**Fig. 2:**
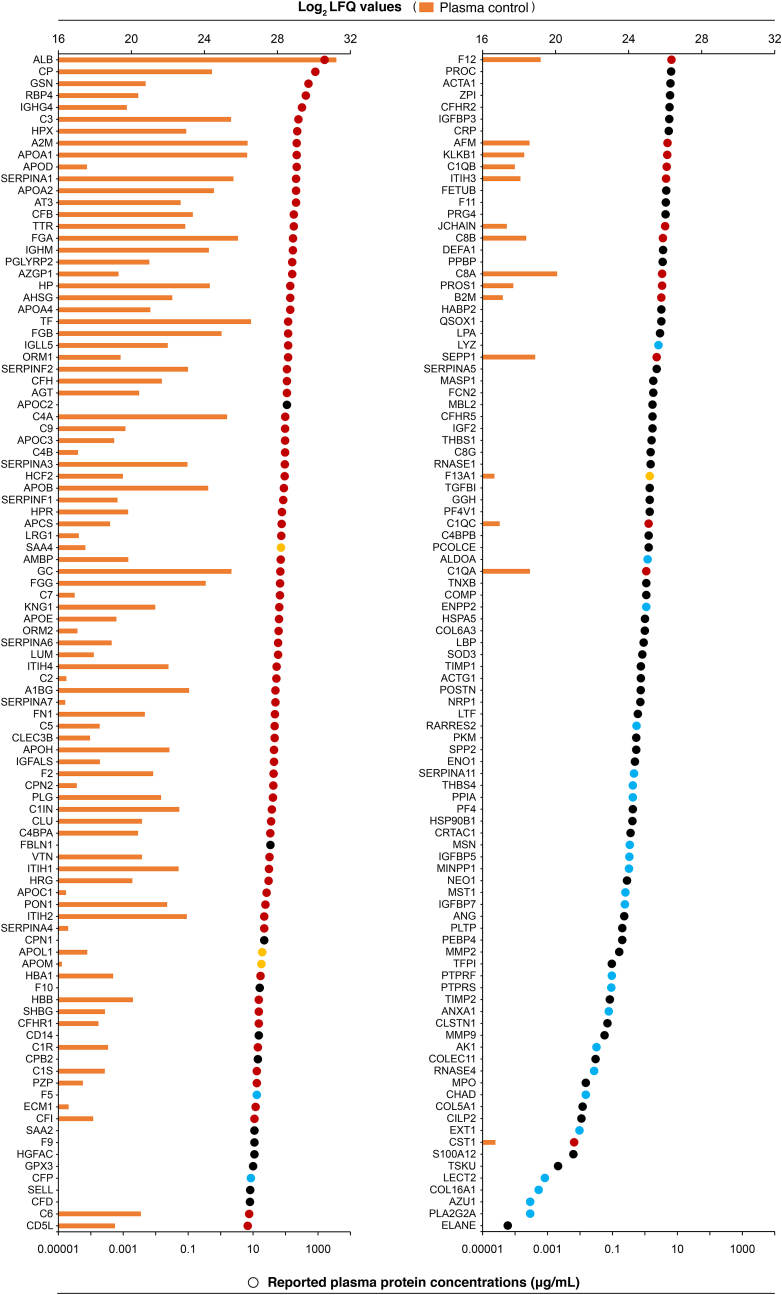
Human plasma proteins identified in this study ranked according to their reported plasma protein concentrations retrieved from the Peptide Atlas [62] and Plasma Proteome Database [63]. Proteins quantified in the unfractionated plasma control are presented as orange bars (*upper axis*) and reported plasma protein concentrations are indicated by dots (*lower axis*). The identified proteins are further categorised (*colouring of dots*) according to their distinct detection: microspheres∗ (*black*), on-microsphere trypsinated (T)-fractions (*blue*), plasma control (*yellow*), and both on microspheres and in plasma control (*red*). In the current work, a total of 241 plasma proteins were confidently identified, of which 198 proteins were reported with plasma concentrations determined by MS spectral counts. Proteins are denoted by gene names.∗Proteins identified in either eluted (E) or E- and T-fractions.

Plasma proteins identified in the current study were assigned their reported concentrations in human plasma using the Peptide Atlas (PA; https://www.proteinatlas.org/humanproteome/blood/proteins+detected+in+ms, accessed Nov. 2021) and the Plasma Proteome Database (PPD; http://www.plasmaproteomedatabase.org, v. 06_2015 retrieved Oct. 2018). The PA (plasma non-glyco 2017 build [62]) presents protein concentrations for 3222 proteins quantified by MS-based plasma proteomics with estimations from spectral counts. The PPD [63] contains published plasma and serum concentrations for 1278 proteins quantified by different methods, wherein plasma protein quantifications by MS spectral counts were only considered. Of the 241 proteins confidently identified in our dataset, plasma concentrations for 198 proteins have been deposited in PA and PPD combined. Proteins that were not reported in the respective databases were mainly immunoglobulin derived. In Fig. 2, the quantified proteins in this study are shown alongside their reported protein concentrations in human plasma. The most abundant proteins (plasma concentrations >1 ​μg/mL) were identified both in the plasma control and on the microspheres, whereas less abundant proteins (plasma concentrations <1 ​μg/mL) were selectively identified on the microspheres. This suggests that distinct subsets of plasma proteins were significantly enriched on the different alginate microspheres. Among the 113 enriched proteins below the cut-off or not detected in plasma, 32 proteins were exclusively detected in the T-fractions of the microspheres. The plasma concentrations for 25 of these proteins are reported and in the range 0.00029–13.0 ​μg/mL (PA or PPD), reflecting a high degree of enrichment on the microspheres.

**Fig. 3:**
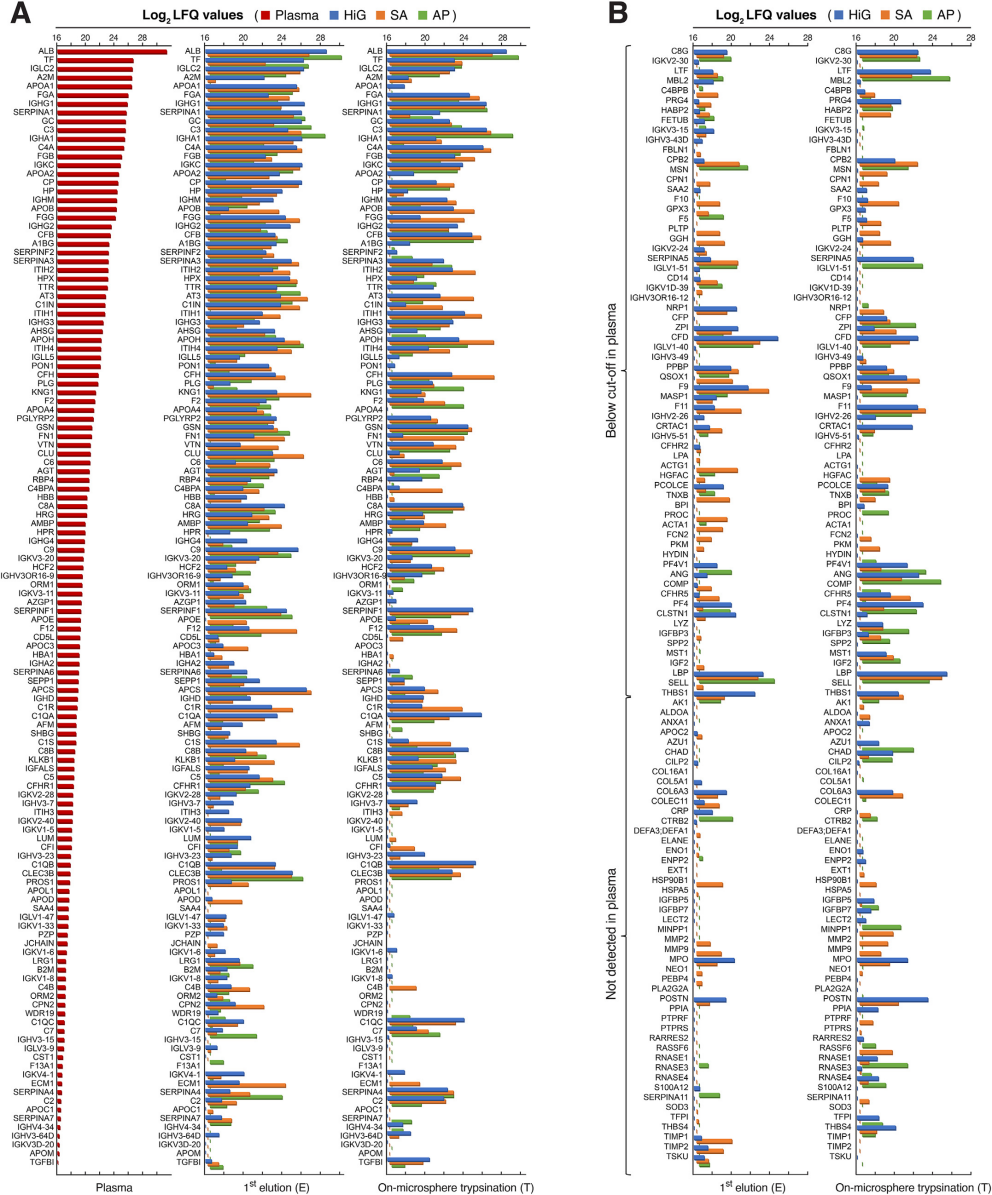
Enrichment of plasma proteins on alginate microspheres after incubation in human lepirudin-plasma (24 ​h). Protein abundances, represented as log_2_ LFQ values, for 241 proteins identified for the microspheres (HiG, SA, AP) and unfractionated plasma control. Microsphere samples comprised separate fractions of eluted (E) and on-microsphere trypsinated (T) proteins. (**A**) Quantified proteins in the plasma control ranked according to their detection level (*descending order*), including the respective protein profiles of the microspheres. (**B**) Enriched proteins on the microspheres, which were below the cut-off or not detected in the plasma control. Proteins are denoted by gene names.

A detailed illustration of the distinct protein adsorption profiles compared to plasma is included in Fig. 3. As shown in Fig. 3A, the protein profiles of the microspheres in terms of relative abundance (log_2_ LFQ values) were different from that of the plasma control. For the majority of the less abundant proteins in the plasma control, a marked enrichment on the microspheres is evident. Proteins below the cut-off or not detected in plasma and quantified on the microspheres are shown in Fig. 3B. Interestingly, among the proteins that were not detected in plasma, many escaped elution in the first step (E-fraction) but were readily quantified in the T-fractions, indicating strong and selective binding to the distinct microsphere types.

**Fig. 4:**
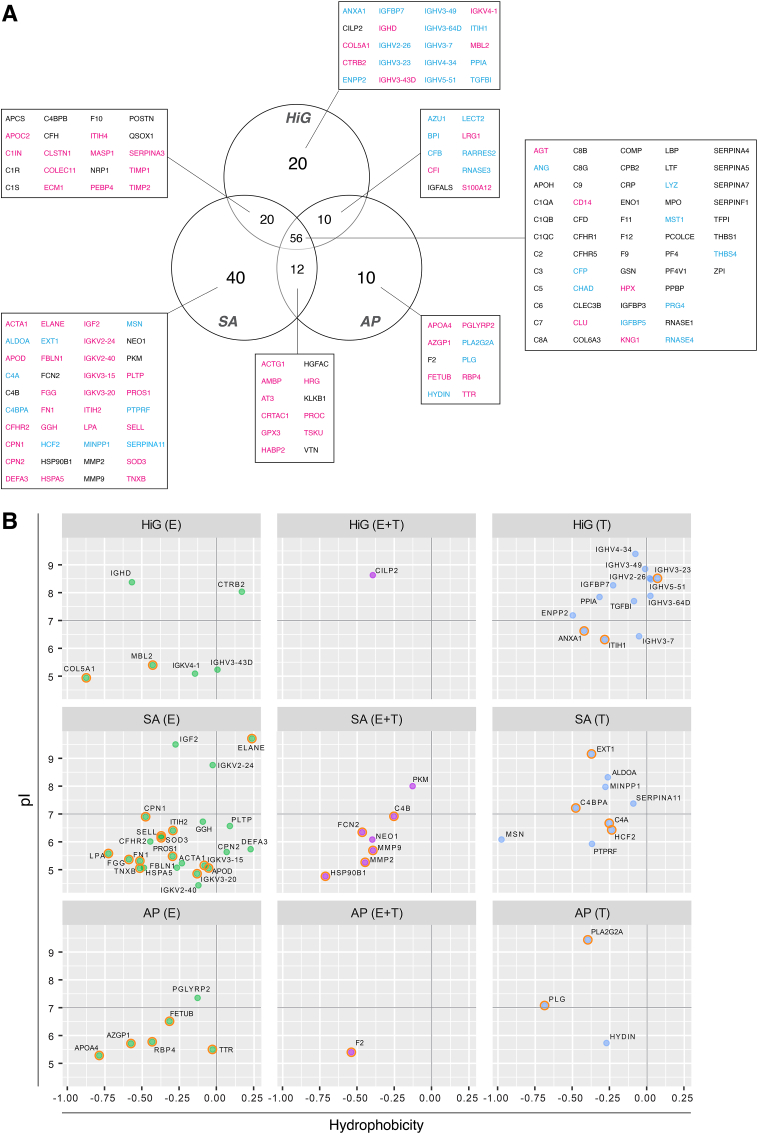
Significantly enriched plasma proteins on alginate microspheres (HiG, SA, AP) after incubation in lepirudin-plasma (24 ​h). (**A**) Venn diagrams showing enriched proteins on the microspheres (E- and T-fractions) compared to the unfractionated plasma control. The statistical significance is given by corrected p-value (FDR) ​≤ ​0.1 and enrichment by ​≥ ​2-fold change. Colour assignments correspond to protein enrichment in the following microsphere fractions: eluted (E)-fraction (*pink*), on-microsphere trypsinated (T)-fraction (*blue*), and both (E ​+ ​T) fractions (*black*). Proteins are listed alphabetically using their gene names. (**B**) Uniquely enriched proteins are characterised by their hydrophobicity score (*x-axis*) and isoelectric point (pI) (*y-axis*). The average hydrophobicity/hydrophilicity of the proteins was calculated using the Kyte-Doolittle method with GRAVY scoring (-2 [hydrophilic] ​< ​0 ​< ​2 [hydrophobic]). pI was calculated along the primary sequence of the protein using the Bjellqvist scale [61]. Heparin- or heparan sulfate-binding proteins [[64], [65], [66], [67]] are encircled in orange.

The number of significantly enriched proteins relative to plasma for microspheres HiG, SA, and AP across the E-/T-fractions were 106, 128, and 88 proteins, respectively (Fig. 4A). SA had the highest number of uniquely enriched proteins (40), compared to HiG (20) and AP (10). Further information on their binding strengths could be derived from the distribution between E- and T-fractions. Interestingly, two proteins were found to be significantly depleted compared to plasma (not detected in either E- or T-fractions); coagulation factor XIII A chain (F13A1) was depleted on all microspheres, and pregnancy zone protein (PZP) was depleted on AP.

Proteins uniquely identified on either HiG, SA, or AP microspheres were further analysed with respect to their physicochemical properties: hydrophobicity/hydrophilicity and charge/isoelectric point (pI) (Fig. 4B). Overall, most of the uniquely adsorbed proteins were hydrophilic in nature (GRAVY score below zero). Also, most acidic proteins (i.e. net negative charge at physiological pH) were found in the E-fractions. In contrast, most basic proteins (i.e. net positive charge at physiological pH) were found in the T-fractions. The latter was especially evident for HiG, in which most proteins observed in the T-fraction were basic and could be explained by opposite charge interactions. Much fewer basic proteins were observed for AP, in agreement with the overall charge neutralisation offered by poly-l-lysine. The number of acidic proteins binding to SA was markedly higher than for HiG. This was somewhat surprising since sulfation increases the number of negatively charged groups on the microbeads. However, most of these proteins have previously been reported to bind heparin or heparan sulfate (Fig. 4B, marked by orange circles). This points to a specific role of the sulfate groups in binding these proteins and likely also contributes to the higher number of uniquely identified proteins on SA.

### 3.3 Different microspheres display distinct adsorption profiles of proteins in the complement and coagulation pathways

**Fig. 5:**
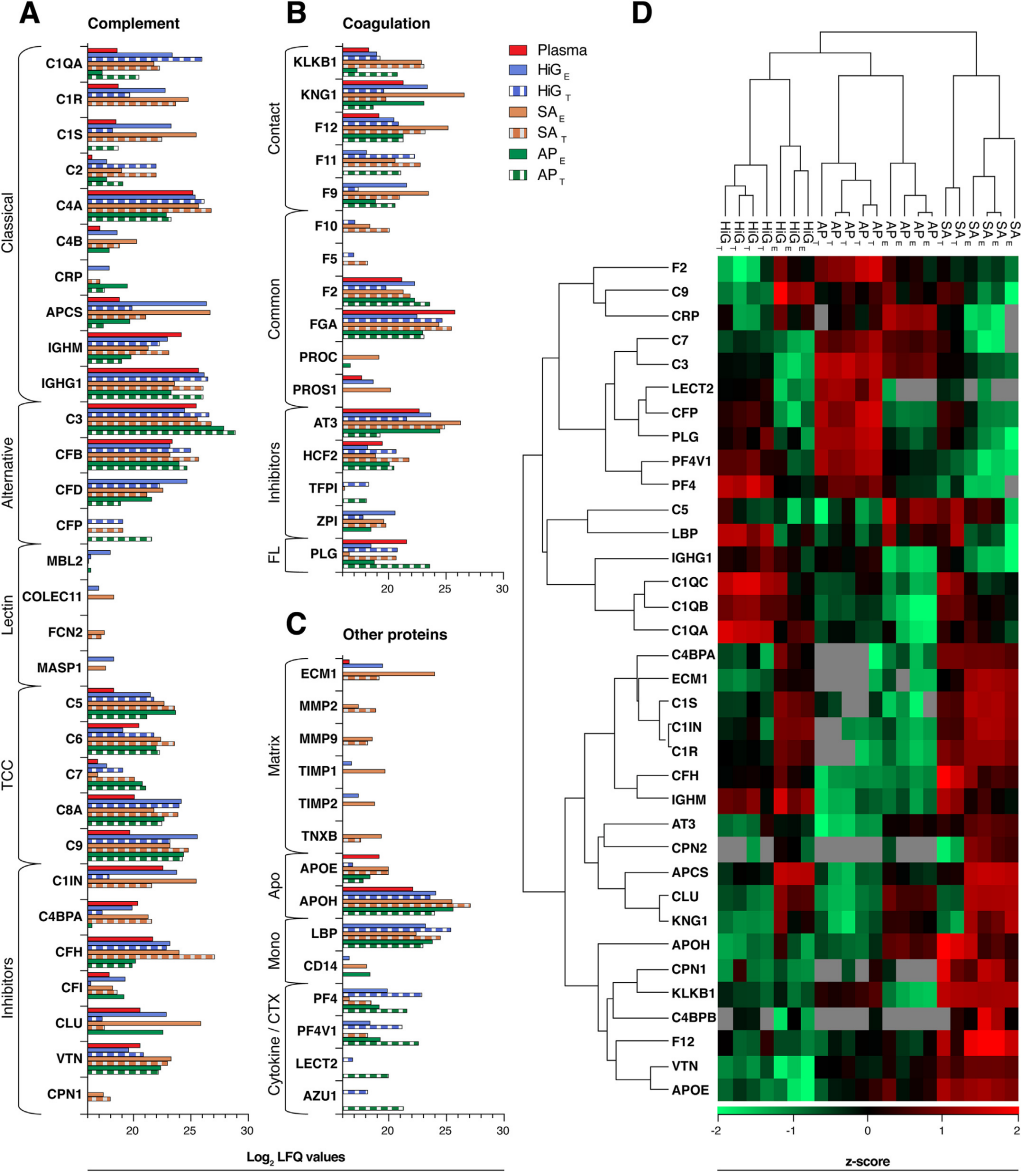
Immune profiling of adsorbed plasma proteins on alginate microspheres. Isolated proteins from eluted (E)- and trypsinated-on-microsphere (T)-fractions of the microspheres (HiG, SA, AP) and the plasma control. (**A**) Adsorbed proteins of the complement system, (**B**) coagulation system, and (**C**) other potentially relevant proteins grouped with their associated pathway or mechanistic function. Protein abundance is given as log_2_ LFQ values, where a difference of 1 equals a 2-fold change. (**D**) Selected adsorbed plasma proteins are presented in the heatmap using z-scored log_2_ LFQ values. The median score is zero (*black*), and the grey colour indicates not detected proteins. Proteins are denoted by gene names. TCC ​= ​terminal complement complex, FL ​= ​fibrinolytic system, Matrix ​= ​extracellular matrix-associated, Apo ​= ​apolipoproteins, Mono ​= ​monocyte associated, CTX ​= ​chemoattractant.

The biological relevance of adsorbed plasma proteins was further analysed to elucidate potential immune profiles for the different microspheres. Ingenuity® Pathway Analysis (IPA) is presented in Table 1, showing the ten most abundant proteins and top canonical pathways identified for the microspheres. The most abundant protein of the plasma control was albumin (ALB), which was also ranked the highest for HiG and AP, but not for SA (ranked third). Besides ALB, protein abundances on the microspheres did not correspond to the unfractionated plasma control, again highlighting the specific protein adsorption to the microspheres. IPA further revealed the top canonical pathways assigned to the bound plasma proteins, including acute-phase response signalling, the complement and coagulation systems, and proteins linked to numerous metabolic pathways such as lipid metabolism (LXR/RXR and FXR/RXR activation). Specifically, bound proteins showed a high degree of overlap with complement and coagulation pathways. Several of these proteins were highly abundant (Table 1) and significantly enriched relative to the plasma control (Fig. 4). Thus, further investigations were made regarding the involvement of complement, coagulation, and other potentially relevant proteins, signifying the immune profiles of HiG, SA, and AP (Fig. 5).

**Table 1:** The upper panel shows the 10 most abundant plasma proteins binding to the different alginate microspheres (E- and T-fractions) with indicated log_2_ LFQ values. The lower panel shows top canonical pathways (IPA analysis) associated with the proteins identified in each fraction from the microspheres and per cent overlap with each pathway. LXR ​= ​Liver X Receptor, RXR ​= ​Retinoid X Receptor, FXR = Farnesoid X Receptor. ∗Number of proteins assigned to pathway (IPA).

10 most abundant plasma proteins *(Log*_*2*_*LFQ values)*							
Plasma control	HiG _E_	HiG _T_	SA _E_	SA _T_	AP _E_	AP _T_	
**ALB***(31.2)*	**ALB *(****28.5)*	**ALB *(****26.7)*	**APCS *(****26.7)*	**CFH *(****27.1)*	**ALB*(****29.6)*	**ALB *(****29.5)*	
**TF***(26.6)*	**APCS *(****26.4)*	**C3 *(****26.6)*	**KNG1 *(****26.7)*	**APOH *(****27.1)*	**C3 *(****27.9)*	**C3 *(****28.9)*	
**IGLC2***(26.4)*	**IGHG1 *(****26.2)*	**IGHG1 *(****26.5)*	**ALB *(****26.5)*	**ALB *(****26.9)*	**GC *(****26.4)*	**IGHG1 *(****26.1)*	
**A2M***(26.4)*	**IGLC2 *(****26.1)*	**C4A *(****26.2)*	**AT3 *(****26.3)*	**C3 *(****26.8)*	**TF *(****26.2)*	**LTF *(****25.1)*	
**APOA1***(26.3)*	**TF *(****26.1)*	**C1QA *(****26.0)*	**CLU *(****25.9)*	**C4A *(****26.8)*	**SERPINA1 *(****25.8)*	**CFB *(****24.7)*	
**FGA***(25.8)*	**IGKC *(****26.0)*	**C1QB *(****25.4)*	**C4A *(****25.7)*	**IGHG1 *(****26.1)*	**APOH *(****25.7)*	**C9 *(****24.3)*	
**IGHG1 *(****25.7)*	**IGHA1 *(****26.0)*	**LBP *(****25.4)*	**C3 *(****25.6)*	**ITIH1 *(****25.8)*	**CLEC3B *(****25.6)*	**ANG *(****24.2)*	
**SERPINA1 *(****25.6)*	**CP *(****25.9)*	**SERPINF1 *(****25.1)*	**IGLC2 *(****25.5)*	**CFB *(****25.7)*	**TTR *(****25.3)*	**GSN *(****24.0)*	
**GC *(****25.5)*	**SERPINA1 *(****25.9)*	**CFB *(****25.0)*	**APOH *(****25.5)*	**FGA *(****25.6)*	**HPX *(****24.9)*	**APOH *(****24.0)*	
**C3 *(****25.5)*	**GC *(****25.9)*	**FGA *(****24.7)*	**IGKC *(****25.5)*	**ITIH2 *(****25.1)*	**IGKC *(****24.5)*	**PLG *(****23.7)*	

**Top canonical pathways***(overlap)*							
24.4%	26.1%	24.4%	25.6%	21.1%	22.2%	19.4%	Acute phase response *(180∗)*
51.4%	62.2%	62.2%	64.9%	59.5%	51.4%	48.6%	Complement system *(37∗)*
42.9%	48.6%	54.3%	57.1%	45.7%	42.9%	42.9%	Coagulation system *(35∗)*
30.6%	29.8%	25.6%	33.9%	19.0%	26.4%	19.8%	LXR/RXR Activation *(121∗)*
29.4%	27.8%	23.0%	29.4%	15.9%	24.6%	18.3%	FXR/RXR Activation *(126∗)*

In Fig. 5A, adsorbed proteins of the complement system are classified according to their associated pathway (classical, lectin, alternative) or function [68,69]. Microbeads HiG and SA were specifically enriched with proteins of the classical and lectin pathways. This was especially evident for the C1 complex (complement C1q with its associated proteases C1r and C1s), involved in the initiation of the classical pathway. C1q comprises subunits C1QA, C1QB and C1QC, which were 152-197-fold enriched on HiG (values given relative to plasma control), 13-140-fold on SA and 2-9-fold on AP, whereas the proteases (C1R, C1S) were only enriched on HiG (17-27-fold) and SA (65-122-fold). In agreement with this, the level of C1q surface-recruiting protein serum amyloid P-component (APCS) was highly pronounced on HiG (>193-fold) and SA (>230-fold), as well as lower-level enrichment of immunoglobulin G1 (IGHG1) and C-reactive protein (CRP). AP had the highest enrichment of CRP, being >3-fold above the other microbeads. The proteins comprising the classical C3 convertase (C4B, C2 [A]), which is activated by the C1 complex, were more prominent on HiG and SA. Proteins of the lectin pathway, including mannose-binding protein C (MBL2), ficolin-2 (FCN2), collectin-11 (COLEC11) and mannan-binding lectin serine protease 1 (MASP1), were also identified on HiG and/or SA but below the detection level in the plasma control. In addition to activators of the classical and lectin pathways, we observed selective enrichment of inhibitors for these pathways on HiG and SA. Plasma protease C1 inhibitor (C1IN), which inhibits the C1 complex and MASPs, respectively, was enriched on SA (7-fold) and HiG (2-fold). C4-binding protein (C4BPA/C4BPB complex), an inhibitor of the classical pathway, was exclusively enriched on SA (>2-fold). The selective enrichment of proteins from the alternative pathway was, however, not as evident on HiG and SA as compared to AP. This pathway is the main driving force of terminal complement activity, involving complement component 3 (C3) and complement factors B (CFB), D (CFD) and properdin (CFP). C3 plays a central role in complement activation, acting as a point of convergence for all three pathways and fuelling the amplification process. CFB and CFD both contribute to the activity of C3. CFP can serve as a local initiator of the pathway by recruiting C3 to the surface and also plays a vital role in stabilising the alternative C3 convertase. AP had the highest enrichment of C3 and CFP, roughly 5-fold above HiG and SA. Notably, CFP was not detected in plasma and was exclusively enriched in the T-fractions, indicating strong binding. Crucial inhibitors of C3 activity include complement factor H (CFH) and I (CFI). CFH was significantly enriched on SA (>44-fold) and to some extent on HiG (>2-fold), while below the plasma control for AP. CFI was enriched (∼2-fold) on all microspheres. Complement components of the terminal complex (TCC; C5, C6, C7, C8, C9) were enriched (2-59-fold) on all microspheres. Inhibitors of TCC assembly, including clusterin (CLU) and vitronectin (VTN), were most pronounced on SA (CLU: 40-fold, VTN: >6-fold). Carboxypeptidase-N (CPN; CPN1/CPN2), which degrades anaphylatoxins (C3a, C5a) to their desArg forms, was distinctively enriched (catalytic subunit CPN1) on SA.

The coagulation protein profiles are summarised in Fig. 5B. Proteins of the contact activation (intrinsic) pathway [70], including plasma kallikrein (KLKB1), kininogen-1 (KNG1), coagulation factors XII (F12), XI (F11) and IX (F9), were all enriched on the microspheres and especially evident on SA. Here F12, which initiates the pathway, was enriched >64-fold on SA compared to >3-fold and >4-fold on HiG and AP, respectively. Proteins of the tissue factor (extrinsic) pathway, including factor III (F3) and VII (F7), were not detected in the dataset. The common coagulation proteins, factors X (F10) and V (F5), were distinct for SA and HiG. Vitamin K-dependent protein C (PROC) that functions as an anticoagulant when activated, including its cofactor vitamin K-dependent protein S (PROS1), were particularly enriched on SA. As part of the clotting process, prothrombin (factor II) is proteolytically cleaved to form thrombin (factor IIa) that converts fibrinogen into fibrin. Prothrombin/thrombin (F2) was most enriched on AP (>5-fold), followed by HiG (2-fold) and SA (<2-fold). Inhibitors of thrombin include antithrombin-III (AT3), which inhibits several coagulation factors, and heparin cofactor 2 (HCF2). Notably, SA showed the highest enrichment of AT3 (>12-fold) and HCF2 (4-fold). Plasmin is generated from the zymogen plasminogen on the surface of fibrin clots as part of the fibrinolytic system, which has also been implicated in complement-independent cleavage of C5 and C3 [69]. Plasminogen/plasmin (PLG) was exclusively enriched in the T-fraction of AP (4-fold).

Fig. 5C displays adsorbed proteins with various biological functions that may be relevant to the PFO outcomes of the microspheres. These proteins include extracellular matrix (ECM)-associated proteins, apolipoproteins, monocyte-associated proteins and cytokines or chemoattractants. For the ECM proteins, ECM protein 1 (ECM1) was extensively enriched on SA (>170-fold) and to some extent on HiG (7-fold), and tenascin-X (TNXB) was distinctively identified on SA. Further, the ECM degrading proteins: matrix metalloproteinase-2 (MMP2) and -9 (MMP9) and neutrophil elastase (ELANE; Table S1) were highly enriched and unique for SA. The tissue inhibitors of metalloproteinase 1 (TIMP1) and 2 (TIMP2), which regulate the MMPs, were exclusively enriched on SA and HiG. Several apolipoproteins (not all shown; see Table S1 for full list of proteins) were either unique or most enriched on SA, including apolipoprotein E (APOE) and H (APOH). Proteins involved in monocyte activation by lipopolysaccharide (LPS) include the LPS-binding protein (LBP) and monocyte differentiation antigen CD14, which were enriched on all three microspheres. The pro-inflammatory cytokines, including platelet factor 4 (PF4), PF4 variant 1 (PF4V1) and leukocyte cell-derived chemotaxin-2 (LECT2), and the chemoattractant azurocidin (AZU1), were enriched above the detection level in plasma and consistently more pronounced or exclusive for HiG and AP.

A selection of the identified proteins is presented in a heatmap (Fig. 5D), showing the relative protein abundances between the three types of microspheres across the E- and T-fractions. Samples from each type of microsphere formed distinct clusters, and the largest dissimilarity in the immune profiles was found between SA and the two other microspheres, HiG and AP. From the cluster analysis of adsorbed proteins, the first level of grouping predominantly separates pro-inflammatory proteins (upper group, encompassing complement mediators and cytokines) from other proteins (lower group, encompassing complement inhibitors and coagulation proteins). Illustratively, proteins of the pro-inflammatory group are clearly most abundant on AP, and proteins of the more anti-inflammatory group are more abundant on SA. HiG represents an intermediate profile between the respective groups. Moreover, the clustering of IGHG1 with C1q (C1QA, C1QB, C1QC) may indicate immunoglobulin-mediated classical complement activation for HiG and SA, whereas the clustering of C1IN with the C1q proteases (C1R, C1S) may indicate the subsequent inactivation of the classical pathway for these microbeads.

### 3.4 Complement and coagulation assessments by CLSM and human whole blood assay

**Fig. 6:**
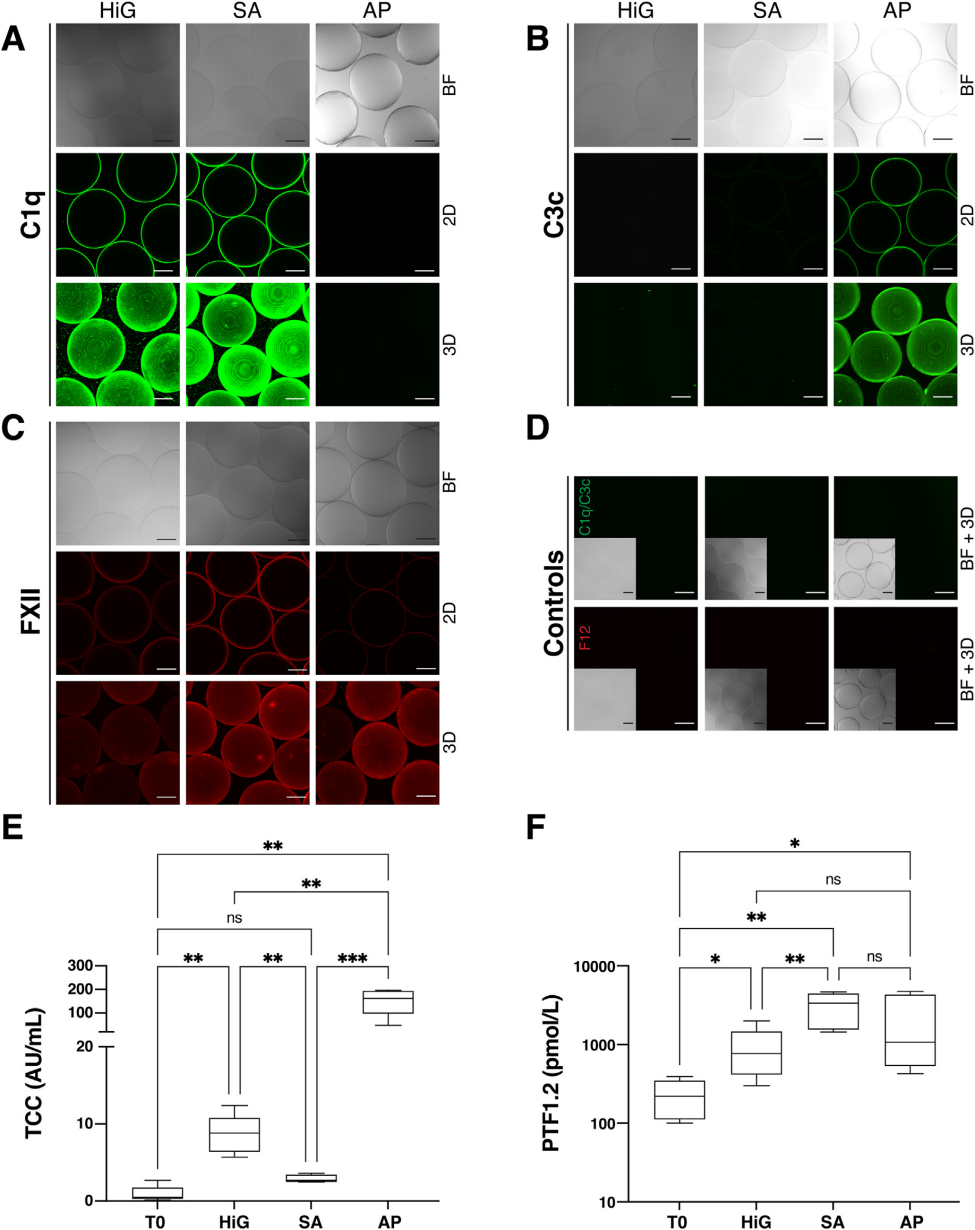
Evaluation of complement and coagulation proteins and responses on/to alginate microspheres. Detection of complement (C1q, C3c) and coagulation proteins (FXII) in human plasma by CLSM (**A-D**) and activation in human whole blood by terminal complement complex (TCC) and prothrombin fragment 1 ​+ ​2 (PTF1.2) (**E, F**). CLSM images show antibody-stained alginate microspheres HiG, SA, and AP after incubation (24 h) in lepirudin-anticoagulated human plasma. Microspheres were FITC-stained (*green*) against complement C1q (**A**) and C3c (**B**) and CF633-stained (*red*) against coagulation FXII (**C**), and non-specific antibody binding was assessed (**D**). Captured images include brightfield (BF), equatorial (2D)-sections and (3D)-projections of z-stacked images. Scale bar: 200 ​μm. Activation of complement by TCC-induction (**E**) and coagulation by PTF1.2 formation (**F**) was assessed after incubation (4 h) in lepirudin-anticoagulated human whole blood. Controls measured (median ​± ​SEM), but not shown, included positive control for PTF1.2 (glass): 759 ​954 ​± ​197 ​581 ​pmol/L, and saline control: 12.9 ​± ​5.2 AU/mL (TCC) and 401 ​± ​169 ​pmol/L (PTF1.2). Data are expressed in box plots with values from five donors. Significant values are given as p ​≤ ​0.05 (∗), p ​≤ ​0.01 (∗∗), p ​≤ ​0.001 (∗∗∗) between indicated sample groups. T0 represents the experimental baseline. (For interpretation of the references to colour in this figure legend, the reader is referred to the Web version of this article.)

The deposition of complement and coagulation proteins on the microspheres was studied by CLSM, and functional responses were assessed in a human whole blood assay (Fig. 6). The binding of C1q was distinct for microspheres HiG and SA (Fig. 6A). Complement C3c, a stable C3-conversion product, was exclusively detected on the surface of AP (Fig. 6B), in accordance with the proteomics data showing a high and selective enrichment of C3 and CFP (stabilises C3-convertases) on these microspheres (Fig. 5A). The binding of FXII was detected for all three microspheres, accumulated in the order SA ​> ​AP ​> ​HiG (Fig. 6C). The non-specific binding of antibodies to the microspheres was negligible (Fig. 6D). On a general note, the protein depositions at the outer layer of the microspheres indicate surface binding rather than passive diffusion into the hydrogels (i.e. absorption). In summary, the CLSM analyses are in agreement with the MS analyses.

In the human whole blood assay, complement and coagulation reactivity was determined by levels of TCC and prothrombin fragment 1 ​+ ​2 (PTF1.2), respectively. Whereas no significant TCC induction was observed for SA, low induction was observed for HiG (p ​< ​0.01) and a strong induction for AP (p ​< ​0.001) compared to baseline (Fig. 6E). The saline control for TCC (not shown) was significantly elevated (p ​< ​0.01) above baseline and SA, whereas not statistically different from HiG and AP. Conversely, the activation of coagulation was most pronounced for SA (p ​< ​0.01), followed by AP and HiG (p ​< ​0.05) compared to baseline (Fig. 6F). The PTF1.2-response for SA as well as for the positive control (glass, not shown) were also significantly (p ​< ​0.01) elevated above the saline control (not shown).

**Fig. 7:**
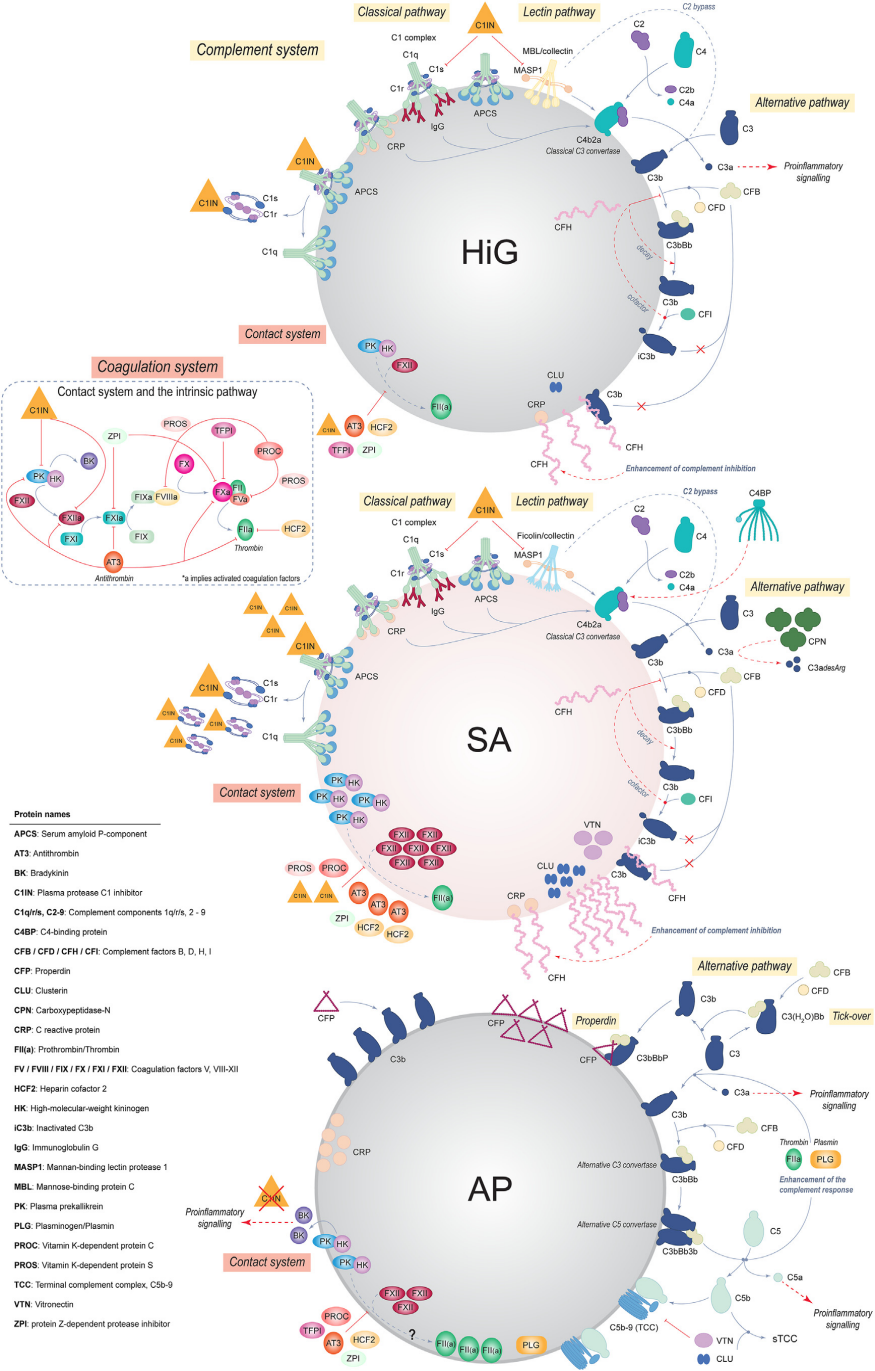
Proposed mechanisms of complement and coagulation responses on the alginate microspheres based on proteomic and CLSM analyses as well as functional data from human whole blood (hWB). The complement and coagulation systems are interconnected protein defence systems [71], consisting of sequential activation of zymogens to active proteinases during inflammatory responses. **The complement system** is activated by three distinct pathways: classical, lectin and alternative, which have been thoroughly described elsewhere [68,69]. HiG and SA are significantly enriched with proteins of both the classical (e.g. C1q and APCS) and lectin pathways (MBL/collectins/ficolins), which may suggest an initial complement activation. However, low levels of C3 indicate a prompt attenuation of this potential complement response through initial regulators. SA is unique in the high abundance of adsorbed complement inhibitors (C1IN, CFH, CFI, C4BP, CPN, CLU, VTN). HiG is enriched with fewer inhibitors (C1IN, CFH, CFI, CLU) and to a lesser extent. HiG, and especially SA, exhibit low terminal complement activity (fluid-phase TCC; sTCC) in hWB, despite being enriched with proteins involved in TCC formation, which suggest inactivation of TCC or adsorption of non-assembled TCC-components. AP displays pronounced enrichment of CFP and C3, indicating complement initiation and propagation through the alternative pathway. Moreover, enrichment of prothrombin/thrombin (FII [a]) and plasminogen/plasmin (PLG) also present the additional possibility of a complement-independent activation of C3 and C5. AP is distinct in the absence or low levels of initial complement inhibitors. This is in accordance with a persisting complement response and subsequent terminal complement activity, shown for these microspheres by the high levels of adsorbed proteins involved in TCC formation and the significant TCC response in hWB. **The coagulation system** is activated through two mechanisms: the extrinsic (tissue factor) pathway (not discussed here) and the intrinsic (contact activation) pathway, which has been described elsewhere [70,[72], [73], [74]]. All the microspheres activate coagulation to some extent, as seen by the functional hWB data. SA has an overall moderate coagulation reactivity but the highest coagulation (prothrombin fragment 1 ​+ ​2) response among the microspheres. Being highly enriched with proteins of the intrinsic pathway, such as FXII, suggests coagulation activation through the contact system. SA is also enriched with numerous coagulation inhibitors (AT3, HCF2, C1IN, PROC, ZPI), which likely diminishes this activation. HiG shares a similar coagulation profile to SA in types of enriched proteins, although at significantly lower abundances and with some variation in adsorbed inhibitors (i.e. PROC, TFPI). Distinctively, AP is enriched with all the contact system proteins, except for C1IN, which potentially allows for pro-inflammatory signalling by BK. Further enrichments include several coagulation inhibitors (AT3, HCF2, PROC, ZPI, TFPI). AP shows the highest enrichment of FII(a) yet distinctively lacks central proteins (FX, FV) of the intrinsic pathway. Hence, the observed coagulation reactivity in hWB indicates tissue factor-dependent initiation [48], closely linked to complement activation.

A graphical summary of proposed complement and coagulation mechanisms for the three types of alginate microspheres is presented in Fig. 7, with a basis on elucidated proteomic immune profiles (Fig. 5) and functional data on complement and coagulation reactivity (Fig. 6). Details are outlined in the figure legend.

## 4 Discussion

Here, we use high-resolution LC-MS/MS to identify distinct protein adsorption profiles for three types of alginate microspheres relevant for immune isolation in cell therapy, which we recently reported exhibit different inflammatory and PFO responses: HiG (low-inflammatory and fibrotic), SA (low-inflammatory and anti-fibrotic), and AP (pro-inflammatory and highly fibrotic) [10]. A total of 236 proteins were differentially adsorbed to the microspheres, and in-depth analyses of the proteins revealed unique immune profiles. In detail, the microspheres showed differences in enriched complement and coagulation factors with inhibitory or activating roles. Of specific importance is the prominent enrichment of inhibitors of complement and coagulation on SA, coinciding with minimal PFO. These findings were supported by functional assessments in human whole blood (hWB) involving total complement and coagulation activation. The current work establishes MS-based quantitative proteomics as a valuable tool, in conjunction with established *in vitro* and *in vivo* methods, to identify potential drivers of inflammation and PFO for alginate-based hydrogels. In the following, we discuss our methodological strategy and the effect of hydrophobicity/hydrophilicity and charge/isoelectric point. Further, we include an in-depth discussion of specific proteins involved in the complement and coagulation cascades that were prominent in the canonical pathway analysis, as well as other proteins that may be relevant in PFO development.

The competitive displacement of adhering surface proteins by other proteins with higher binding affinities is commonly referred to as the Vroman effect [37,75]. Consequently, the protein adsorption behaviour of a material depends on the composition of the protein solution and the exposure time. In the current study, we chose a strategy that preserved the reactive complement proteins and coagulation factors in human plasma, which sets an important premise for the subsequent protein displacements. To distinguish between weaker and stronger bound proteins, we chose to employ a two-step detachment procedure (primary elution [E] and secondly on-microsphere trypsination [T]). The T-fraction represents the proteins with the overall strongest binding, which is supported by the many proteins below the detection level in plasma selectively identified in the T-fraction. The highly abundant plasma proteins were predominantly identified in the E-fraction. The enrichment of proteins from plasma was evident for all the studied microspheres. However, the number of different proteins was highest on the anti-fibrotic SA and lowest on the pro-fibrotic AP. This indicates that the number of proteins by itself is not a driver of the host response, but rather the type of proteins and their capability to react with the material surface, as will be discussed in later sections.

Hydrogels constitute a unique class of hydrophilic materials, comprising water-swollen networks of hydrophilic polymers. Their protein adsorption behaviour is thus not directly comparable to that of other non-hydrogel hydrophilic materials, such as functionalised solid surfaces that are more widely described in the literature ([76] and references therein). The three types of hydrogel microspheres in this study primarily differ in their functional groups, which also dictate the surface charges. The alginate (present in all the microspheres) is negatively charged at physiological pH due to the presence of carboxylate groups (-COO^-^), while the sulfated alginate (in SA) carries additional negative charges from the chemically introduced sulfate groups (-SO_4_^2-^) at C2 and/or C3 [24]. Complexation of the alginate hydrogel with poly-l-lysine (PLL; AP), which contains positively charged amino groups (-NH_3_^+^) at physiological pH, decreases the net negative charge at the capsule surface of AP [5]. The number of significantly enriched proteins increased with the negative charge of the microspheres (SA ​> ​HiG ​> ​AP) (Fig. 4A), pointing to charge interactions as important drivers for the observed protein binding. There was a trend towards a more positive charge of the proteins in the T-fraction, supporting that opposing charge interactions play an important role in promoting the strongest binding to the microspheres. The high number of negatively charged proteins uniquely binding to the microspheres may be explained by positively charged surface patches that could dictate their binding despite an overall net negative charge [38]. One such example is the known sugar-binder inter-alpha-trypsin inhibitor heavy chain 1 (ITIH1). This protein contains a positively charged lysine cluster aligned on top of a β-sheet on the protein surface (PDB: 6FPY) [77], which might explain the strong association to HiG. The inclusion of sulfated alginate in the alginate microbeads (SA) resulted in an overall increase in adsorbed proteins as well as uniquely enriched proteins in the E-fraction. Notably, the majority (>63%) of the negatively charged proteins binding to SA have previously been reported to bind heparin and/or heparan sulfate [[64], [65], [66], [67]]. Heparin and heparan sulfate are sulfated glycosaminoglycans (GAGs) that interact with a wide range of proteins in the regulation of different biological processes such as the immune response or response to wounding [64]. The structure-function properties of sulfated alginate have been associated with those of heparin and heparan sulfate [24,78], and sulfated alginate has previously been shown to bind several heparin-binding proteins [24,26].

Alginate hydrogels have water-swollen, porous structures (∼2% [w/v] polymer concentration) that could allow proteins to penetrate below the microsphere surface [79]. Coating alginate microbeads with PLL decreases the pore size of the microspheres [80], which could contribute to the lower number of proteins on AP. Previous studies on the surface topography of alginate microspheres using atomic force microscopy (AFM) revealed similar average surface roughness for Ca- or Ba-alginate microbeads and alginate-PLL-alginate microspheres [81]. In a recent AFM study, we showed that the inclusion of oxidized alginate in soft alginate hydrogels leads to an increase in surface roughness [82]. Like oxidized alginate, sulfated alginate also contributes poorly to the ionic network of the hydrogel [83,84]. Thus, higher surface roughness could potentially be expected for SA when compared to HiG. However, the effect of topography as a driver for protein adsorption in the studied microspheres remains elusive.

Complement is considered a key contributor to biomaterial-induced host responses [41]. The coagulation cascade may influence the host response by direct activation through the contact pathway or through crosstalk with the complement system [71]. Ingenuity pathway analysis (IPA) identified the acute-phase response and the complement and coagulation cascades as the top enriched canonical pathways for all three microspheres. However, the protein adsorption does not necessarily reflect a biological response, since the acute-phase effectors which include most of the complement and coagulation proteins depend on distinct conformational changes to elicit their biological functions [41,85]. Thus, we interpret the proteomics data (Fig. 5) alongside the functional data in hWB with assessments of total complement and coagulation activation (Fig. 6). In agreement with our findings, initial *in vivo* adsorption of acute-phase proteins was also reported for fibrotic PEG hydrogels explanted from mice [27]. This supports the significance of active proteolytic cascades in the early phases of the host response and fibrotic tissue development, as well as the relevance of using lepirudin plasma *in vitro*.

In brief, the complement system consists of an intricate network of effectors and regulators. Driven by conformational changes, complement activation could be initiated through the alternative, classical, or lectin pathways. This culminates in the formation of convertases, which in turn generate the complement effectors, including opsonins (e.g. C1q, C3b), anaphylatoxins (e.g. C3a, C5a), and terminal complement complex (TCC) [68,69]. The potent biological effects of these effectors are tightly regulated by inhibitors, consisting of cellular receptors or soluble humoral factors that also bind to GAGs on cell surfaces [68,86,87]. In the current study, SA showed insignificant terminal complement activation (TCC formation) in hWB, which represents the functional endpoint of complement activation. This conforms with the enrichment of complement inhibitors in the proteomic analysis, including C1 inhibitor (C1IN), factor H (CFH), C4-binding protein (C4BP), clusterin (CLU), vitronectin (VTN), and carboxypeptidase-N (CPN). Interestingly, the catalytic subunit of CPN, which serves to mitigate the pro-inflammatory, chemotactic signalling of the anaphylatoxins C3a and C5a [69], was exclusively enriched on SA. The abundant enrichment of CFH on SA is intriguing as it is involved in the inhibition of complement at the level of C3. It is acknowledged that CFH can bind directly to polyanions such as sulfated GAGs and sialic acid on host cells [85]. It has also been demonstrated that C1IN directly binds to heparin [88] which exhibits properties similar to sulfated alginate. The enrichment of complement inhibitors to SA (containing sulfated alginate) is likely biologically significant, as shown by the low TCC response and could at least partly be related to a direct binding of these factors. In addition, low levels of C3 in both proteomics (enriched >2-fold from plasma) and CLSM analyses, together with a pronounced enrichment of CFH (>44-fold) for SA, indicates that the balance of activating and inhibiting proteins is important for the biological outcome. This is further apparent when comparing to AP, which showed low levels of the complement inhibitors while the highest deposition of C3 and properdin (CFP). This further coincides with a significant TCC response in hWB. The abundant plasma protein C3 is crucial for downstream complement activation, where CFP serves to stabilise the C3 protein in its active form (C3 convertase) [68]. Thus, the data indicates that the C3 convertase is built at the surface of AP, with further downstream activation of the complement cascade leading to the formation of TCC. Previously, we have shown that the binding of C3 to the surface of AP leads to the adhesion of leukocytes *in vitro* with subsequent secretion of pro-inflammatory cytokines [15,16]. In agreement with our previous work, the proteomics study by Wang et al. on human serum proteins adsorbed to PEG hydrogels revealed the binding and activation of C3, where the inactivation of C3 (incubation in C3-inactivated serum) led to a reduction in the attachment of human monocytes *in vitro* [20].

In the functional assay (hWB) herein, HiG induced a low TCC response yet significantly elevated above that of SA. The proteomic analysis may provide insight into the subtle differences in inflammatory potentials of the microspheres, showing that the enriched complement inhibitors were significantly less abundant on HiG compared to SA (Fig. 7). Intriguingly, both microspheres showed an extensive enrichment of C1q (classical pathway), as well as being enriched with proteins of the lectin pathway, which could indicate an initial complement recognition. However, if this recognition leads to complement activation it is likely subdued at an early stage, in accordance with enriched inhibitors (C1IN), lower-level enrichment of C3, minimal C3c deposition verified by CLSM and, importantly, low TCC induction in the functional hWB assay. In support, the cluster analysis of bound proteins showed a potential inactivation of C1 complex (C1q/C1r/C1s) by grouping its inhibitor (C1IN) with the C1q-associated proteases (C1r, C1s). C1IN is known to bind these proteases, forming an inactive covalent complex that dissociates from C1q and hinders downstream complement activation [69]. Interestingly, C1q was more enriched on HiG whereas C1IN was more enriched on SA, which might explain the slightly lower complement activation of SA in hWB. It has previously been shown that sulfated polysaccharides (e.g. sulfated GAGs and dextran sulfate) potentiate C1IN activity [89]. The anti-inflammatory effects of C1IN encompass the regulation of the classical and lectin complement pathways, as well as the coagulation (contact pathway) and fibrinolytic systems [86].

In the assessment of the total coagulation activation in hWB (protrombin factor 1 ​+ ​2; PTF1.2), we found an elevated response for SA. The proteomics data showed a marked enrichment of the initiators of the contact pathway on SA, including coagulation factor XII (FXII), plasma kallikrein (KLKB1) and high-molecular-weight kininogen (KNG1), thus pointing to an initiation through the contact pathway. Still, the coagulation response of SA is considered moderate in comparison to the glass control, which is a prominent activator of FXII [48]. This could be explained by the enrichment of C1IN, which also regulates the contact pathway. In addition, SA was enriched with several coagulation inhibitors, especially antithrombin-III (AT3) and heparin cofactor 2 (HCF2). This finding points to a similarity to heparin that mediates its anticoagulation effect through the binding of AT3 [90].

In the following sections, we discuss the proteomics data in connection with the previously reported PFO outcomes and focus on the poorly understood differences in PFO between HiG and SA, the former being prone to PFO as opposed to the latter exhibiting minimal PFO *in vivo* [10]. Their elucidated immune profiles present similar protein identities, except for a few regulatory proteins, yet they display distinct protein abundances. These subtle differences might tip the balance toward a PFO response as seen for HiG. In detail, SA was unique in its abundant and strong association to a multitude of complement inhibitors, which were less prominent on HiG. Importantly, this includes the binding of complement factor H (CFH) that was profoundly more enriched on SA compared to HiG and, contrastingly, not enriched on AP. The abundant enrichment of CFH is particularly interesting as it serves a protective role against complement-targeted destruction in the host [68], and CFH enrichment strategies have previously been used to improve the biocompatibility of biomaterials [91]. In addition, an extensive enrichment of serum amyloid-P component (APCS) was found on HiG and SA. APCS is involved in several aspects of innate immunity, including inhibiting the differentiation of fibrocytes and pro-fibrotic macrophages, and has been proposed as a potential anti-fibrotic therapeutic [92]. The strong binding of IgG (e.g. IGHG1) and other immunoglobulins was consistently more pronounced on HiG. The classical pathway, mediated by the action of C1q and its numerous pattern recognition molecules, is strongly initiated by IgG or IgM [68]. Cluster analysis of the proteomics data grouped IgG with C1q (Fig. 5D), which was most prominent for HiG and could indicate a relatively higher degree of C1q activation. As discussed above, the clustering of C1IN with the C1q-associated proteases was most prominent for SA and could point to a relatively larger degree of C1q inactivation. Thus, the potential activation state of C1q might prove relevant for the PFO outcomes of HiG (fibrotic) and SA (anti-fibrotic). Complement proteins (such as C3) play a central role in fibrotic tissue development [17,19], and we recently demonstrated the *in vivo* deposition of C3 on highly PFO-prone AP microspheres [10]. In the proteomics study herein, AP displayed the highest enrichment of C3 and, intriguingly, the acute-phase protein C-reactive protein (CRP). CRP is involved in classical complement activation [68] and has further been shown to promote cell-mediated responses with potential recruitment of inflammatory cells [93]. Romero-Gavilán et al. found that CRP, as well as C1q, was most abundant on titanium-based implants that were prone to fibrotic responses, wherein the authors postulate that the ratio of complement activators and inhibitors may determine the *in vivo* outcome [30].

The fibrinolytic system is known to restrict the propagation of blood clots by dissolving fibrin during wound healing processes. In our previous *in vivo* experiments [10], the accumulation of fibrin(ogen) on the microspheres was found to coincide with PFO, where SA exhibited a significantly lower degree of PFO and fibrin(ogen) deposition than HiG and AP. In the current study, specific regulatory factors of fibrinolysis [94], involving the activation of plasminogen to form the active fibrinolytic enzyme plasmin, could indicate that SA has a higher inherent potential for fibrinolysis. The levels of the plasminogen activators FXII and KLKB1 were markedly higher on SA, and the plasmin inhibitor alpha-2-macroglobulin (A2M) was strikingly more depleted, compared to HiG and AP (Supplementary Fig. S2).

The significantly enriched heparin-binding proteins which were unique for the anti-fibrotic SA include the matrix metalloproteinases (MMPs [MMP2, MMP9]), neutrophil elastase (ELANE) and tenascin-X (TNXB). The ECM degrading enzymes MMP2 and MMP9 have been found to inhibit, and also promote, fibrosis in different fibrotic disease models [95,96]. Interestingly, the MMPs and their regulators (TIMPs) were enriched on SA, while only the latter was enriched on HiG. The intricate balance between MMPs and TIMPs and their potential roles in fibrosis might prove relevant for biomaterial-mediated fibrotic responses and, in effect, the fibrotic outcome for SA and HiG. The neutrophil-derived protease ELANE modulates innate immunity and inflammation, where excessive activity has been shown to hamper phagocytic recognition and clearance through the digestion of opsonins and opsonin receptors [97]. The ECM glycoprotein TNXB plays an important role in ECM architecture and tissue integrity, which has been shown to have anti-adhesive properties [98]. Several apolipoproteins involved in lipid metabolism, among others [99], were detected on the microspheres, which were consistently more pronounced on SA compared to HiG and AP. Interestingly, the apolipoproteins E (APOE) and H (APOH; β2-glycoprotein 1) have been reported to have anti-fibrotic effects [100,101].

In summary, the previous sections have highlighted proteins that may have an impact on the PFO responses seen *in vivo* [10], with a particular focus on HiG and SA. Sulfated alginate reduces the fibrotic host response towards empty and cell-containing microbeads compared to high G alginate [3,10], demonstrating the significance of using biomaterials with low inflammatory potential in cellular therapies that depend on the free diffusion of oxygen and nutrients to encapsulated cells. In this study, the binding of inhibitors (e.g. C1IN and CFH) and heparin-binding proteins to SA are striking. Recruiting CFH to biomaterial surfaces has previously been shown to attenuate biomaterial-mediated inflammatory responses [91], thus underscoring the clinical relevance of surface-associating proteins in terms of biocompatibility. Importantly, as the proteomic analysis does not provide information on the activation state of the proteins, it needs to be supported with functional studies. Here, we focused on the proteolytically active cascades of complement and coagulation in human whole blood. The proteomics data serves to deepen our understanding of the mechanisms of activation by providing insight into the binding of activators and inhibitors to the biomaterial surface. This culminates in the biological effects seen as complement or coagulation activation *in vitro* or fibrotic tissue development *in vivo*.

## 5 Conclusion

The current study is the first to document the adsorption of human plasma proteins on alginate hydrogel microspheres using LC-MS/MS-based proteomics. In conjunction with functional data of complement and coagulation activation in human whole blood, the proteomic analysis signifies a physiologically relevant method for elucidating protein-based immune profiles of alginate hydrogel materials. Utilising lepirudin-based plasma enables the investigation of proteolytic activable cascades of the complement and coagulation systems, which are critical activators of the host immune system. Protein profiling revealed novel details on the selective protein binding to the different alginate-based materials, and that could help explain their different PFO outcomes shown in previous studies. The abundant binding of complement and coagulation inhibitors to SA conforms to a low-inflammatory and anti-fibrotic profile. Moderate levels of inhibitors to HiG conform to a low-inflammatory but pro-fibrotic profile. In contrast, the enrichment of complement activators in combination with low amounts of inhibitors on AP conforms to a pro-inflammatory and pro-fibrotic profile. The current work presents a step forward in understanding the underlying mechanisms of the inflammatory and PFO responses towards alginate-based microspheres. SA possibly evades fibrosis through the extensive binding of acute-phase inhibitors. Heparin-binding proteins enriched on SA may have additional anti-fibrotic effects. Ultimately, the method and results presented in this study can serve as tools to tailor novel biocompatible alginate materials intended for cell-based therapies as well as other therapeutic or diagnostic applications.

## Credit author statement

**Abba E. Coron**: Conceptualisation, Methodology, Validation, Formal analysis, Investigation, Writing – original draft, Writing – review & editing, Visualisation. **Davi M. Fonseca**: Conceptualisation, Methodology, Validation, Investigation, Writing – review & editing. **Animesh Sharma**: Conceptualisation, Methodology, Validation, Formal analysis, Resources, Writing – review & editing. **Geir Slupphaug**: Conceptualisation, Methodology, Validation, Resources, Writing – review & editing, Funding acquisition. **Berit L. Strand**: Conceptualisation, Methodology, Validation, Resources, Writing – review & editing, Supervision, Project administration, Funding acquisition. **Anne Mari A. Rokstad**: Conceptualisation, Methodology, Validation, Investigation, Resources, Writing – review & editing, Supervision, Project administration, Funding acquisition.

## Funding

This work was supported by the 10.13039/100009123Norwegian University of Science and Technology (NTNU), Faculty for Natural Science, The Liaison Committee for Education, Research, and Innovation in Central Norway (Regional Health Authority, Samarbeidsorganet) under grant 46056819, CEMIR NFR grant 223255, 10.13039/100009123NTNU Health project “Tailored biomaterials with reduced immune responses**”,** and the Chicago Diabetes Project (www.chicagodiabetesproject.org). PROMEC is funded by the Faculty of Medicine at 10.13039/100009123NTNU and 10.13039/501100004590Central Norway Regional Health Authority. PROMEC is a member of the National Network of Advanced Proteomics Infrastructure (NAPI), which is funded by the RCN INFRASTRUKTUR-program (295910).

## Declaration of competing interest

The authors declare that they have no known competing financial interests or personal relationships that could have appeared to influence the work reported in this paper.

## Data Availability

Data will be made available on request.

## Appendix A Supplementary data

Fig. S1 + S2 Fig. S1 + S2
