# Supplementary material for: MS-proteomics provides insight into the host responses towards alginate microspheres
Source: Mater Today Bio. 2022 Nov 11;17:100490. doi: 10.1016/j.mtbio.2022.100490 (PMC9676213; doi:10.1016/j.mtbio.2022.100490)
Supplement: Fig. S1 + S2 [file mmc1.pdf]

Supplementary data

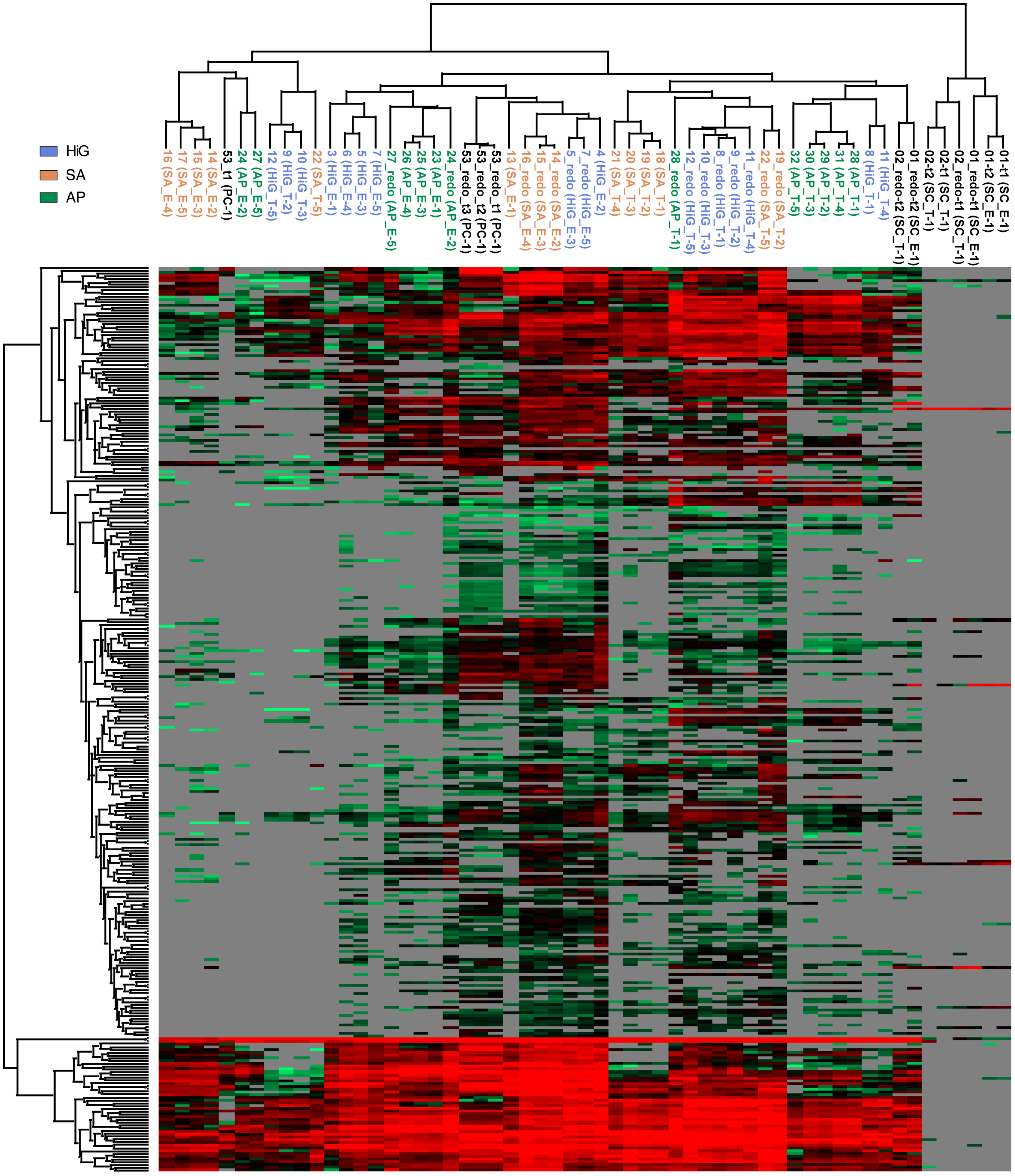

Figure S1. Heatmap of LC-MS/MS-analysed samples (raw data) and reanalysed replicates (redo). Samples include isolated proteins from plasma-incubated microspheres (HiG, SA, AP; eluted [E]- and trypsinated-on-microsphere [T]-fractions), unfractionated plasma control (PC), and saline controls (microspheres incubated in saline; SC). Median $\log_2$ LFQ values using z-score from -2 (green) to +2 (red), where black equals the median value of 0. Grey colour represents not detected proteins.

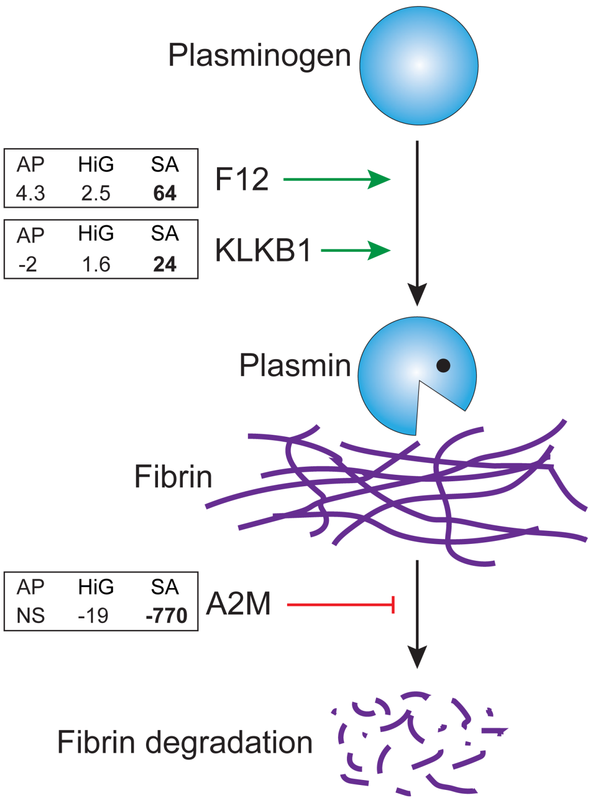

Figure S2. Factors potentially mediating increased fibrinolytic capacity of SA microbeads. Coagulation factor XII (F12) and plasma kallikrein (KLKB1) stimulate plasmin activation and alpha-2-macroglobulin (A2M) inhibits plasmin activity. Enrichment and depletion of these factors from the eluted microsphere fractions are indicated under each microsphere as fold-change values compared to the plasma control. NS; not significant.
