# Supplementary material for: MS-proteomics provides insight into the host responses towards alginate microspheres
Source: Mater Today Bio. 2022 Nov 11;17:100490. doi: 10.1016/j.mtbio.2022.100490 (PMC9676213; doi:10.1016/j.mtbio.2022.100490)
Supplement: Table S1 [file mmc2.pdf]

Table S1 Identified proteins by high-resolution LC-MS/MS

Gene names	ONE Protein ID	Plasma CTR	HiG (E)	SA (E)	AP (E)	HiG (T)	SA (T)	AP (T)	Saline CTR (E)	Saline CTR (T)	Peptides	Score
ALB	P02768	31,2	28,5	26,5	29,6	28,7	26,9	29,5	15,5	15,5	126	323,31
TF	P02787	26,6	26,1	21,9	26,2	23,1	23,7	23,4			89	323,31
IGLC2	P0DOY2	26,4	26,1	25,5	23,8	23,2	22,4	21,1			16	323,31
A2M	P01023	26,4	22,1	16,8	15,4	18,2	18,3	13,3			77	323,31
APOA1	P02647	26,3	25,4	25,4	24,5	17,8	14,7	14,7			50	323,31
FGA	P02671	25,8	22,5	24,4	23,0	24,7	25,5	23,1			67	323,31
IGHG1	P01857	25,7	26,2	23,6	23,3	26,5	26,1	26,1		13,3	30	323,31
SERPINA1	P01009	25,6	25,9	24,9	25,8	21,6	18,1	20,3			51	323,31
GC	P02774	25,5	25,9	22,8	26,4	22,6	22,6	23,4			50	323,31
C3	P01024	25,5	24,5	25,6	27,9	26,6	26,8	28,9		18,3	179	323,31
IGHA1	P01876	25,4	26,0	24,8	21,5	21,2	21,5	17,2			20	284,67
C4A	P0C0L4	25,2	25,4	25,7	22,9	26,2	26,8	23,3			136	323,31
FGB	P02675	24,9	22,2	22,6	20,5	23,2	25,1	21,2			57	323,31
IGKC	P01834	24,8	26,0	25,5	24,5	24,0	23,5	23,0	16,4		19	323,31
APOA2	P02652	24,5	23,6	22,3	17,6	18,8	15,6	14,9			9	323,31
CP	P00450	24,4	25,9	25,4	20,2	21,2	22,9	22,1			72	323,31
HP	P00738	24,3	23,9	23,1	19,3	18,1	17,5	16,5			35	279,64
IGHM	P01871	24,2	23,0	21,3	19,8	22,3	23,1	19,0	15,1		30	323,31
APOB	P04114	24,2	18,4	23,3	17,0	23,0	25,0	14,5			188	323,31
FGG	P02679	24,1	24,3	25,5	21,7	19,5	23,9	14,0			40	323,31
IGHG2	P01859	23,5	24,8	21,4	21,9	23,5	22,3	22,0			24	159,07
CFB	P00751	23,4	23,2	23,2	24,0	25,0	25,7	24,7			119	323,31
A1BG	P04217	23,2	23,3	22,5	21,4	18,4	14,9	16,3			31	235,71
SERPINF2	P08697	23,1	22,2	22,8	21,4	17,0	13,7	18,2			23	123,53
SERPINA3	P01011	23,1	24,9	25,4	23,0	22,0	20,5	22,4	15,3		36	323,31
ITIH2	P19823	23,0	22,9	24,5	21,1	22,9	25,1	19,4			46	323,31
HPX	P02790	23,0	24,7	25,2	24,9	19,1	18,5	20,7			40	323,31
TTR	P02766	23,0	23,4	23,1	25,3	20,9	13,4	13,8			15	234,57
SERPINC1 (AT3)	P01008	22,7	23,7	26,3	24,5	21,6	24,9	19,3			46	323,31
SERPING1 (C1IN	P05155	22,6	23,8	25,5	12,8	17,9	21,6	13,5			25	323,31
ITIH1	P19827	22,6	21,9	23,5	20,6	24,2	25,8	22,4			48	323,31
IGHG3	P01860	22,4	21,6	19,7	18,7	23,0	22,3	21,1			29	318,21
AHSG	P02765	22,2	23,1	21,9	22,8	19,1	13,9	19,6			17	217,99
APOH	P02749	22,1	24,1	25,5	25,6	23,6	27,1	24,0			32	323,31
ITIH4	B7ZKJ8	22,0	23,4	24,6	19,6	20,4	22,4	18,2			54	323,31
IGLL5	B9A064	22,0	19,5	19,0	16,5	17,2	15,8	15,8			14	27,445
PON1	P27169	22,0	22,5	22,5	17,9	16,8	15,5	13,0	13,2		19	323,31
CFH	P08603	21,7	23,2	24,0	20,2	22,9	27,1	19,9			102	323,31
PLG	P00747	21,6	18,5	16,6	18,8	20,8	20,7	23,6			94	323,31
KNG1	P01042	21,3	23,4	26,6	23,1	19,6	19,8	18,7			40	323,31
F2	P00734	21,2	22,3	21,3	22,3	19,8	21,9	23,6			51	323,31
APOA4	P06727	21,0	21,3	21,8	22,3	14,5		14,5			42	323,31
PGLYRP2	Q96PD5	21,0	23,3	22,8	21,9	20,6	21,1	20,7			26	323,31
GSN	P06396	20,8	23,0	23,2	24,2	24,6	24,7	24,0			78	323,31
FN1	P02751	20,7	21,0	23,9	14,2	17,6	23,9	11,2	14,7	12,6	96	323,31
VTN	P04004	20,6	19,6	23,3	22,4	20,9	23,0	22,2		10,9	35	314,67
CLU	P10909	20,6	22,9	25,9	22,6	17,3	17,5	15,9			34	323,31
C6	P13671	20,5	19,1	22,4	22,0	21,8	23,6	22,3			55	323,31
AGT	P01019	20,4	23,4	22,8	22,1	19,4	16,5	21,0			18	218,11
RBP4	P02753	20,4	20,7	20,0	21,5	19,7	15,3	16,2			17	323,31
C4BPA	P04003	20,4	19,9	21,3	16,4	17,3	21,6	12,6			25	323,31
HBB	P68871	20,1	20,2	19,4	13,2	14,8	16,5		17,6	17,7	11	88,65
C8A	P07357	20,1	24,2	21,8	22,7	24,0	23,9	22,5			37	323,31
HRG	P04196	20,1	20,8	22,3	21,1	19,7	19,7	17,4			30	323,31
AMBP	P02760	19,8	20,3	23,6	22,1	19,8	22,0	19,0			29	323,31
HPR	P00739	19,8	18,5	16,0	14,3	16,5	13,3				24	103,93
IGHG4	P01861	19,8	20,3	17,5	18,3	19,2	18,3	18,0			19	43,759
C9	P02748	19,7	25,6	23,2	24,4	23,2	24,8	24,3			51	323,31
IGKV3-20	P01619	19,6	21,5	21,0	18,6	18,6	18,2	15,4			5	112,79
SERPIND1 (HCF2	P05546	19,5	18,2	18,9	20,1	20,7	21,8	20,5			33	301,43
IGHV3OR16-9	A0A0B4J2B5	19,4	18,7	17,3	16,3	19,7	17,6	18,3	19,1	20,2	4	323,31
ORM1	P02763	19,4	19,9	20,2	20,2	14,4	12,3	17,1			9	75,256
IGKV3-11	P04433	19,4	19,4	19,7	16,9	16,6	15,2	14,0			6	25,106
AZGP1	P25311	19,3	20,2	18,7	21,9	16,9	13,3	13,1			22	137,71
SERPINF1	P36955	19,2	24,4	23,5	24,5	25,1	24,4	22,3			45	323,31

APOE	P02649	19,2	15,0	20,0	18,4	16,9	20,0	17,8			19	323,31
F12	P00748	19,2	20,5	25,2	21,3	20,9	23,2	21,3			29	323,31
CD5L	O43866	19,1	17,3	17,1	16,6	15,6	17,4				15	77,552
APOC3	P02656	19,1	17,8	20,1	15,9						5	69,974
HBA1	P69905	19,0	16,8	17,5	13,1	13,3	16,4	12,3	18,3	16,7	5	23,52
IGHA2	A0A0G2JMB2	19,0	18,9	18,1	15,3	12,7	14,1				17	23,6
SERPINA6	P08185	18,9	20,3	18,4	19,8	17,3	14,5	18,2			13	88,664
SEPP1	P49908	18,9	21,6	19,7	19,1	17,8	17,5	12,5			6	33,795
APCS	P02743	18,8	26,4	26,7	19,7	19,9	21,1	17,4			19	323,31
IGHD	P01880	18,8	20,6	18,6	15,0	19,8	19,5	13,5			12	71,291
C1R	P00736	18,7	22,8	24,8	14,2	19,7	23,7	13,7			42	323,31
C1QA	P02745	18,6	23,4	21,8	17,3	26,0	22,3	20,5			12	57,811
AFM	P43652	18,6	19,8	17,4	15,3	13,7	15,1	17,1			27	217,24
SHBG	P04278	18,6	18,5	18,1	17,5	15,4	12,3	15,0			11	67,47
C1S	P09871	18,5	23,3	25,5	13,6	18,2	22,5	18,7	17,3	15,6	40	323,31
C8B	P07358	18,4	20,2	21,1	21,8	24,6	22,8	22,8			53	323,31
KLKB1	P03952	18,3	19,0	22,9	17,3	19,3	23,1	20,8			40	323,31
IGFALS	P35858	18,3	20,5	20,1	16,9	20,8	21,9	19,3			28	268,44
C5	P01031	18,3	21,5	22,7	23,7	21,8	23,6	21,2			87	323,31
CFHR1	Q03591	18,2	20,6	19,9	21,0	21,1	20,9	20,4			21	216,08
IGKV2-28	A0A075B6P5	18,1	19,2	17,2	13,8	12,7					5	18,661
IGHV3-7	P01780	18,1	18,9	16,5	15,0	19,1	17,9	16,8			6	75,729
ITIH3	Q06033	18,1	18,4	14,8		14,3	17,3	11,1			19	105
IGKV2-40	A0A087X0Q4	18,0	19,7	19,4	14,6	13,8	12,5	12,9			5	80,444
IGKV1-5	P01602	17,9	17,9	15,2	16,0	16,0	13,9	13,9			3	14,83
LUM	P51884	17,9	20,7	19,1	18,8	15,9	16,6	13,2	15,1		10	147,43
CFI	P05156	17,9	19,3	18,2	19,2	16,3	18,6	15,3			32	323,31
IGHV3-23	P01764	17,8	18,6	16,4	15,1	19,9	17,1	15,7			6	284,74
C1QB	P02746	17,8	23,2	22,9	17,2	25,4	24,9	21,0			11	323,31
CLEC3B	P05452	17,7	25,0	24,7	25,6	22,9	23,5	22,3			22	323,31
PROS1	P07225	17,7	18,7	20,2	14,7	14,3	14,7	15,6			20	323,31
APOL1	O14791	17,6	15,1	15,4		15,4	15,0	12,7			5	22,261
APOD	P05090	17,6	16,7	19,5	15,8	14,2	9,9				14	124,84
SAA4	P35542	17,5	15,3	15,8		13,9	14,8	13,4			7	29,245
IGLV1-47	P01700	17,5	18,1	17,8	15,9	16,7	15,2	13,9			3	15,851
IGKV1-33	P01594	17,4	17,8	18,0	15,8	12,8	13,4				3	65,912
PZP	P20742	17,3	17,9	14,0		14,5	14,1				13	23,81
JCHAIN	P01591	17,3	15,0	16,9	10,1						5	21,83
IGKV1-6	A0A0C4DH72	17,2	18,0	16,7	15,8	17,0	15,5	14,3			4	18,703
LRG1	P02750	17,1	19,5	19,3	20,4	13,6		13,3			12	189,71
B2M	P61769	17,1	18,2	17,3	17,9	14,2	12,5				6	9,4252
IGKV1-8	A0A0C4DH67	17,1	18,2	17,0	15,3	16,5	15,2	13,9			3	26,166
C4B	P0C0L5	17,1	18,6	20,3	17,9	15,2	18,8	14,8			136	42,244
ORM2	P19652	17,0	17,2	17,5	18,0			14,0			8	74,497
CPN2	P22792	17,0	19,1	21,8	17,1	14,8	13,4				15	96,507
WDR19	Q8NEZ3	17,0	17,3	16,0	17,6	15,2		17,9			5	2,6593
C1QC	P02747	16,9	19,9	19,1	16,2	24,2	23,0	18,2			10	323,31
C7	P10643	16,9	17,7	16,9	20,8	19,1	20,1	21,1			42	323,31
IGHV3-15	A0A0B4J1V0	16,9	15,8	13,9	12,8	16,1	15,7	15,4			8	8,2822
IGLV3-9	A0A075B6K5	16,8	17,2	16,3	13,9	14,0	13,8	13,6			3	132,75
CST1	P01037	16,7	15,6	15,2	17,4		14,1	14,2			2	2,4064
F13A1	P00488	16,6									2	2,9792
IGKV4-1	P06312	16,6	20,0	17,3	16,3	17,8	14,2	13,9			5	19,396
ECM1	Q16610	16,6	19,5	24,0	12,6	15,0	19,2	13,0			34	277,14
SERPINA4	P29622	16,5	18,5	20,3	23,5	22,4	22,8	22,6			29	323,31
C2	P06681	16,4	17,7	19,0	17,7	22,0	22,0	19,1			49	196,18
APOC1	P02654	16,4	15,1	16,5	13,7	12,5	13,4				3	7,9344
SERPINA7	P05543	16,4	17,7	18,5	18,1	15,4	14,8	18,1			12	235,49
IGHV4-34	P06331	16,3	15,6	14,2	13,8	17,6	15,2	17,2			5	52,641
IGHV3-64D	A0A0J9YX35	16,2	17,4	15,4	14,6	18,5	17,0				4	4,5554
IGKV3D-20	A0A0C4DH25	16,2	14,7	14,5	10,9	10,7	10,5	10,8			3	2,0103
APOM	O95445	16,2	15,6	15,9	13,7		11,8				6	14,409
TGFBI	Q15582	16,0	16,6	17,1	17,3	20,5	19,5	17,4			25	195,61
C8G	P07360	15,9	19,4	18,8	19,3	22,3	21,9	22,0			15	323,31
IGKV2-30	P06310	15,9	16,4	15,3	12,6	14,5	13,4	13,5			4	6,4533
LTF	P02788	15,8	17,9	18,2	18,5	23,6	21,4	25,1			61	323,31
MBL2	P11226	15,7	18,0	16,1	16,3	16,3	15,8				6	49,005
C4BPB	P20851	15,7	14,9	18,2		16,8	17,5	16,8			4	187,15
PRG4	Q92954	15,7	16,5	17,5	16,6	20,5	19,2	19,1			18	70,318

HABP2	Q14520	15,6	16,5	17,3	17,5	15,4	19,2	16,0			29	323,31
FETUB	Q9UGM5	15,6	17,1	14,7	16,7			16,1			5	29,102
IGKV3-15	P01624	15,5	18,0	16,9	14,6	15,1	15,1	13,9			4	6,8286
IGHV3-43D	P0DP04	15,5	16,8	15,3		15,9	15,1	15,0			4	20,763
FBLN1	P23142	15,4	16,0	16,4				15,4			8	26,617
CPB2	Q96IY4	15,4	17,0	20,4	21,0	19,9	22,0	20,8			18	323,31
MSN	P26038	15,4		14,3		16,0	18,8	16,1			21	107,86
CPN1	P15169	15,3	13,5	17,4	15,0	13,7	18,0	15,2			12	156,4
SAA2	P0DJI9	15,2	16,6	16,1	14,2	17,0		9,3			8	123,87
F10	P00742	15,2	14,4	18,4	15,1	17,1	20,1	15,4			16	145
GPX3	P22352	15,0	15,0	17,2	18,5	16,8	11,6	12,5			12	36,227
F5	P12259	14,9		15,4		17,0	18,2	15,9			19	80,109
PLTP	P55058	14,9	15,2	18,4		13,1	18,1				6	43,918
GGH	Q92820	14,8	15,2	18,9	14,8	16,6	19,2	12,9			11	57,096
IGKV2-24	A0A0C4DH68	14,8	17,0	17,0	14,3	15,3	13,6	14,6			3	8,4387
SERPINA5	P05154	14,8	17,7	20,3	19,9	21,9	16,0	22,3			19	323,31
IGLV1-51	P01701	14,7	16,5	15,2	13,9	13,7	11,6	12,5			3	9,4083
CD14	P08571	14,7	16,6	18,1	18,4	14,3	12,8	13,9			9	68,424
IGKV1D-39	P04432	14,6	15,9	16,5	15,4	14,0	13,2				3	34,782
IGHV3OR16-12	A0A075B7B8	14,6	14,9	12,0	11,2	15,7	13,2	16,6			2	11,259
NRP1	O14786	14,5	20,4	19,1		14,5	18,5	13,1			22	279,22
CFP	P27918	14,3	15,2	15,5	15,8	19,1	19,1	21,6			9	105,42
SERPINA10 (ZPI)	Q9UK55	14,2	20,6	19,6	18,5	17,8	19,8	15,5			24	99,206
CFD	P00746	14,1	24,7	22,6	21,6	22,3	21,2	18,9			11	235,95
IGLV1-40	P01703	14,1	16,1	15,6	14,3	14,2	12,3	13,4			2	6,5873
IGHV3-49	A0A0A0MS15	14,1	15,0	15,2	13,1	16,6	16,6	14,3			6	9,2861
PPBP	P02775	14,0	19,8	20,3	19,1	19,1	19,5	18,7			11	323,31
QSOX1	O00391	14,0	15,5	19,7	15,2	21,1	22,2	15,6			27	268,84
F9	P00740	13,9	21,6	23,5	18,9	17,4	21,0	20,6			28	323,31
MASP1	P48740	13,9	18,3	17,6		14,3	14,4				10	39,273
F11	P03951	13,8	18,1	20,6	15,2	22,3	22,8	21,1			36	323,31
IGHV2-26	A0A0B4J1V2	13,8	17,0	15,1		17,9	14,8	11,7			2	3,9119
CRTAC1	Q9NQ79	13,8	17,6	18,6	16,9	21,7	17,5	17,1			12	39,098
IGHV5-51	A0A0C4DH38	13,7	15,4	14,5	12,5	16,1	15,6	15,3			3	14,13
CFHR2	P36980	13,6	16,6	16,4	15,7		14,7	12,5			15	80,927
LPA	P08519	13,5		16,3	13,6			12,0			5	51,673
ACTG1	P63261	13,5	15,9	20,2	17,6	11,7	12,6	12,3	17,9	17,6	22	266,96
HGFAC	Q04756	13,3	12,2	16,8	12,9	15,4	19,1	17,5			11	213,89
PCOLCE	Q15113	13,2	19,0	16,9	17,6	19,2	18,6	18,7			24	256,87
TNXB	P22105	13,2	15,0	19,4		14,2	17,6				22	220,33
BPI	P17213	13,1	13,9	14,1	14,3	16,7	13,1	18,7			12	113,55
PROC	P04070	12,8	13,5	19,2	16,7		12,0	13,9			16	49,321
ACTA1	P68133	12,8	14,6	18,7	15,9			13,1		16,4	14	8,1876
FCN2	Q15485	12,6	14,9	17,5	12,1	13,3	17,2				7	35,371
PKM	P14618	12,5		16,7		14,2	18,1	13,8			12	160,42
HYDIN	Q4G0P3	12,4	13,1	12,6	14,0	13,8	14,5	17,4			3	1,8359
PF4V1	P10720	12,2	18,4	15,9	19,3	21,2	18,2	22,6			9	173,33
ANG	P03950	12,1	17,3	15,5	14,9	22,4	20,3	24,2			14	143,4
COMP	P49747	12,1	16,3	17,5	15,3		14,2	17,9			12	217,9
CFHR5	Q9BXR6	12,0	16,5	18,3	15,3	19,4	21,3	16,3			27	236,32
PF4	P02776	11,9	19,9	16,6	19,2	22,9	18,5	21,6			8	87,38
CLSTN1	O94985	11,8	20,3	16,9		17,0	15,2				22	172,59
LYZ	P61626	11,8	14,6	14,5	14,5	18,6	18,4	20,8			10	252,24
IGFBP3	P17936	11,8	15,4	16,4	14,0	17,2	18,2	18,8			13	50,004
SPP2	Q13103	11,6	13,5	13,3	16,1	15,6					7	218,28
MST1	P26927	11,6	13,2	15,8	12,1	19,0	19,5	19,9			36	223,04
IGF2	P01344	11,0	13,3	16,7	12,4	11,7	14,4				6	85,192
LBP	P18428	11,0	23,2	22,4	23,8	25,4	24,5	23,0			21	323,31
SELL	P14151	10,9	15,0	16,6			15,6				5	25,921
THBS1	P07996	10,5	22,3	18,9	18,2	20,3	20,5	17,7			55	323,31
POSTN	B1ALD9		19,3	17,3	14,7	23,4	20,0	11,5			19	144,42
TIMP1	P01033		16,8	19,7	13,3		13,5				7	13,493
COLEC11	Q9BWP8		17,0	18,3	14,8						9	104,33
TIMP2	P16035		17,4	18,8	14,7		14,2				7	13,082
HSP90B1	P14625		14,8	18,7		14,5	17,7		19,3	19,6	17	317,14
MMP9	P14780		14,7	18,6			18,2			17,2	13	55,295
CRP	P02741		17,9	13,9	19,5	15,6	17,1	17,5			6	35,174
S100A12	P80511		16,6		18,1	14,2					4	5,4409
COL6A3	P12111		19,4	18,2	15,2	19,7	20,5	16,3			31	181,85

MPO	P05164	20,2	18,1	13,2	21,3	19,1	16,0		33	213,68
CHAD	O15335	12,4	13,6	14,2	19,7	16,9	19,1		10	182,96
THBS4	P35443	13,1	15,2		20,0	17,8	17,3		8	19,382
MINPP1	Q9UNW1		14,1	13,7	13,4	19,5	14,1		9	68,383
RASSF6	Q6ZTQ3		13,3	12,7		19,4	13,0		2	1,7106
AZU1	P20160	13,1	11,9	14,8	18,2	15,1	21,3		10	64,514
RNASE1	P07998	14,1	14,1	16,9	18,1	16,9	20,7		7	19,157
LECT2	O14960	13,7	13,8	13,4	16,9	15,8	20,0		4	13,763
RNASE4	P34096	11,7	15,1	14,7	18,2	16,9	18,4		6	59,46
TFPI	P10646	12,8	16,2	13,6	18,3	14,5	18,1		10	128,45
MMP2	P08253	15,6	17,4			18,9	15,0		18	120,31
PPIA	P62937	12,1	15,4	15,2	18,2	12,3	14,4		11	63,081
ANXA1	P04083	14,5			17,3	14,3	15,8		6	24,578
PTPRF	P10586	13,1	15,8		14,6	17,4			12	17,591
RARRES2	Q99969	11,3	12,0	11,5	16,6	15,1	17,4		8	26,342
IGFBP7	Q16270	12,3	10,5	11,9	17,4	13,2	14,9		9	15,191
IGFBP5	P24593	12,8	15,0	12,8	17,7	16,2	17,7		14	39,731
TSKU	Q8WUA8	17,1	17,2	17,1	15,6		15,6		4	27,213
ALDOA	P04075	13,1	13,6	13,6	12,6	17,0	11,8		9	20,482
ENO1	P06733	14,1	16,2	16,3	16,6	15,7	15,1		20	98,824
NEO1	Q92859		16,5			16,2			9	12,769
PEBP4	Q96S96	16,0	16,5	15,3	14,6	14,6		14,6	8	23,198
CILP2	Q8IUL8	16,4	12,4		16,2	13,4			7	13,935
SOD3	P08294	15,2	16,1			12,7			6	91,838
RNASE3	P12724	12,6	12,6		16,4	16,1	16,9		6	83,593
AK1	P00568	13,8	14,8			16,4			5	14,5
ENPP2	Q13822		15,6		16,9	15,7	15,7		10	15,93
EXT1	Q16394		13,7			16,4			5	13,151
HSPA5	P11021	12,5	16,1	13,0		15,9			13	114,93
COL5A1	P20908	16,8	14,5						4	11,077
ELANE	P08246	13,3	16,1	16,0	14,1	15,2	15,3		4	26,81
CTRB2	Q6GPI1	16,2	15,9		16,0	14,6			4	96,021
PTPRS	Q13332	12,0	11,9		12,6	16,1			4	9,8358
SERPINA11	Q86U17	14,7	14,4	12,0	14,2	17,0	9,1		4	9,9041
DEFA3	P59666	15,3	16,3		16,0	13,5	13,3		3	12,784
COL16A1	Q07092				14,5	11,5	16,1		3	4,0774
APOC2	P02655	16,3	16,5			11,3			2	6,2688
PLA2G2A	P14555				12,5		16,0		2	52,003
